# Single-cell sequencing reveals cell heterogeneity and aberrantly activated pathways associated with microvascular invasion in hepatocellular carcinoma

**DOI:** 10.3389/fcell.2025.1449624

**Published:** 2025-01-29

**Authors:** Jianwei Cui, Fanyi Zeng, Ming Tang, Shiwu Yin

**Affiliations:** ^1^ Department of Interventional Vascular Medicine, Hefei Hospital Affiliated to Anhui Medical University, The Second People’s Hospital of Hefei, Hefei, Anhui, China; ^2^ The Fifth Clinical College of Medicine, Anhui Medical University, Hefei, Anhui, China

**Keywords:** single-cell RNA-sequencing, hepatocellular carcinoma, microvascular invasion, MARCKSL1 gene, prognostic signature

## Abstract

**Introduction:**

Hepatocellular carcinoma (HCC) is the most common primary liver cancer, with microvascular invasion (MVI) identified as a major predictor of early recurrence. However, the intratumor cellular heterogeneity of MVI, the identification of pertinent biomarkers, and the role of intercellular signalling interactions in MVI progression are unclear. This study aims to explore these aspects using single-cell transcriptomic analysis.

**Methods:**

The present study utilized single-cell transcriptomic data from public databases to conduct an in-depth transcriptome analysis of tumour tissues and adjacent nontumor tissues from five patients with hepatocellular carcinoma, with a particular focus on samples from three patients exhibiting microvascular invasion. Bioinformatics tools were employed to analyze gene expression patterns and signalling pathways.

**Results:**

The findings indicated that MVI-positive malignant cells activate multiple signalling pathways to facilitate invasion and metastasis. Specific malignant cell subtypes strongly associated with MVI were identified, exhibiting distinctive gene expression patterns related to proliferation, invasion, and metabolic reprogramming of tumour cells. Further analysis revealed that the laminin and VEGF signalling pathways are crucial for remodelling the tumour microenvironment and angiogenesis associated with MVI. The MARCKSL1 gene was predominantly expressed in MVI-positive malignant cells and may contribute to MVI progression by interacting with the PTN signalling network. Additionally, MARCKSL1 is linked to tumour resistance to multiple anticancer drugs.

**Discussion:**

This study sheds light on the molecular characteristics and functional heterogeneity of MVI-associated malignant cell subpopulations. The single-cell transcriptome and bioinformatics analyses provided insights into the mechanisms driving MVI, potentially aiding the development of targeted diagnostic and therapeutic strategies. Future research should further validate the role of MARCKSL1 in MVI progression and explore its potential clinical applications.

## 1 Introduction

Hepatocellular carcinoma (HCC) is the third leading cause of cancer-related death worldwide, with the number of deaths and diagnoses expected to increase by more than 55% globally by 2040 (Marrero et al., 2018; Rumgay et al., 2022). Currently, the primary treatments are liver resection and liver transplantation. However, the recurrence rates remain high after treatment, with 5-year recurrence rates of 70% and 35% after liver resection and liver transplantation, respectively (Xu et al., 2019). In recent years, significant attention has been given to the role of microvascular invasion (MVI) in HCC. MVI, defined as the invasion of tumour cells into the spaces between vascular endothelial cells, including portal veins, hepatic arteries, and lymphatic vessels, is an independent risk factor for early postoperative recurrence and poor prognosis in HCC patients (Gouw et al., 2011). Notably, for patients with a solitary small HCC lesion less than 5 cm in diameter, the presence of MVI significantly reduces recurrence-free survival (RFS) and overall survival (OS) rates (Sheng et al., 2020; Hong et al., 2021; Xiong et al., 2022; Sumie et al., 2008). Thus, there is an urgent need for more specific molecular biomarkers with prognostic and therapeutic significance.

The rapid advancement of single-cell RNA sequencing (scRNA-seq) technology in recent years has revolutionized the understanding of cellular heterogeneity in various pathological tissues (Ramachandran et al., 2019; Kuppe et al., 2021). ScRNA-seq has led to significant discoveries in liver cancer research. Studies have shown that tumour-associated macrophages (TAMs) in liver cancer are closely linked to poor patient prognosis, and they have identified critical genes in the inflammatory response of TAMs, such as SLC40A1 and GPNMB (Ma et al., 2019; Zhang et al., 2019). Additionally, scRNA-seq has been used to map various immune cell subpopulations within liver cancer tissues, including T cells and dendritic cells. Each subpopulation plays a unique role in the liver cancer microenvironment. For example, LAMP3-positive dendritic cells mediate immune suppression, while TREM2-positive TAMs inhibit the infiltration of CD8^+^ T cells into tumour tissue (Zhang et al., 2019; Zheng et al., 2017; Tan et al., 2023).

Despite these findings, a comprehensive understanding of the expression profiles of malignant cells in hepatocellular carcinoma, particularly during the progression of MVI, is lacking, and their specific roles in tumours are unclear. The present study investigated the expression profiles of malignant cells in hepatocellular carcinoma, systematically classified these cells, and detailed the cellular heterogeneity associated with MVI, as well as the molecular biological features of specific malignant subpopulations. A machine learning approach was used to construct a prognostic model based on signature genes of malignant cells, which not only enhanced the prognostic utility of the signature gene but also identified a previously unreported molecule, namely, MARCKSL1. Further studies indicated that MARCKSL1 may promote the development of MVI through interactions with the PTN signalling network. The present findings suggested that MARCKSL1, a potential therapeutic target for hepatocellular carcinoma and MVI progression, may be crucial for improving therapeutic strategies and clinical outcomes, particularly for patients with MVI.

## 2 Materials and methods

### 2.1 Data collection

TCGA-LIHC data were sourced from UCSC Xena (https://xenabrowser.net/datapages/), and scRNA-seq data were obtained from the GEO database (accession number GSE242889). The dataset included samples from five patients diagnosed with HCC, three of whom had MVI, and two without MVI. For each patient, both HCC tissue and adjacent non-tumor tissue were collected. After stringent quality control, we analyzed a total of 42,070 cells, which included 4,070 high-quality sequenced cells. The dataset underwent single-cell sequencing using the Illumina NovaSeq 6,000 platform.

### 2.2 Data processing

For preprocessing, quality control, normalization, and dimensionality reduction clustering of the single-cell data, we employed Seurat v4.3.0. Potential doublets were identified and removed using DoubletFinder v2.0.3. The quality control criteria included the expression of at least 400 genes per cell and a mitochondrial gene threshold of 20%. Subsequent normalization, identification of highly variable genes, and dimensionality reduction clustering were performed using Seurat’s default parameters and standard workflow. Harmony v1.2.3 was utilized to integrate data from different samples. Cell cluster naming was conducted by aggregating marker genes from the literature and through manual annotations. The FindAllMarkers function was employed to identify differentially expressed genes between cell subgroups using the Wilcoxon test, with selection criteria of an adjusted p-value <0.05 and |log2FC| > 0.5.

### 2.3 Cell type identification

We performed differential expression analysis of all genes within cell clusters using Seurat’s FindAllMarkers function to identify marker genes for each cluster. Criteria for identifying marker genes included an adjusted *P* value <0.05, expression percentage >0.25, and |log2[fold change (FC)]| > 0.25. Subsequently, different cell clusters were identified and annotated using the singleR packages, based on the compositional patterns of the marker genes. These annotations were manually validated and corrected with reference to the CellMarker database.

### 2.4 Cell-cell communication

The ‘CellChat’ R package v1.1.3 was utilized to infer cellular communication within the tumor microenvironment based on receptor-ligand interactions (Gupta et al., 2021). This involved counting the number of links and collecting communication probabilities to calculate communication networks. The interaction time and the total strength of interactions between two arbitrary cell populations were visualized. Scatter plots were drawn to illustrate the primary sender (source of signals) and receiver cells (targets) within a two-dimensional space. This visualization helps identify the most significant contributors to the outgoing or incoming signals among a group of immune cells. We employed a pattern recognition approach, the global communication model, to discern how various immune cell types and signaling pathways operate in concert.

### 2.5 Pseudotemporal trajectory analysis

Cellular pseudotemporal trajectories were constructed using the Monocle two algorithm, an R package developed by Qiu et al. for single-cell trajectories (Huang et al., 2019). This algorithm employs machine learning techniques to reduce the high-dimensional expression spectrum into a low-dimensional space, organizing it into trajectories with branching points. Dynamic expression heatmaps were constructed using the plot_pseudotime_heatmap function. Integrated Machine Learning-Based Approach to Derive Feature Signatures: We integrated up to 10 machine learning algorithms, including Random Survival Forest (RSF), Elastic Net (Enet), Lasso, Ridge, Stepwise Cox, CoxBoost, Partial Least Squares Regression for Cox (plsRcox), Supervised Principal Components (SuperPC), Generalized Augmented Regression Model (GBM), and Survival Support Vector Machine (survival-SVM). Based on these approaches, a consensus model was generated. In total, 101 algorithm combinations were executed to match the predictive model based on the leave-one-out cross-validation (LOOCV) framework. The TCGA-LIHC dataset was divided into training and test datasets.

### 2.6 Immune infiltration assessment

We utilized the CIBERSORT algorithm to quantitatively assess the level of immune cell infiltration in patients with pancreatic adenocarcinoma (PAAD), exploring differences in cell abundance between high-risk and low-risk patient groups (Malumbres and Barbacid, 2009). Additionally, we calculated and analyzed the Pearson correlation between immune cell abundance and risk scores. To further delineate potential differences in immune function, we applied enrichment scores obtained from single-sample gene set enrichment analysis (ssGSEA) (He et al., 2021). Subsequently, we used the Wilcoxon test to compare immune function between the high-risk and low-risk groups.

### 2.7 Statistical analysis

All statistical analyses and data visualization were conducted using R software (version 4.1.3). Pearson’s correlation coefficient was used to assess the relationship between two continuous variables. For quantitative data, values were compared between subgroups using two-tailed, unpaired Student’s t-test or one-way analysis of variance (ANOVA) with Tukey’s multiple comparison test. A *P*-value <0.05 was considered statistically significant.

## 3 Results

### 3.1 Single-cell sequencing and cytotyping of cancerous and paracancerous tissues

**FIGURE 1:**
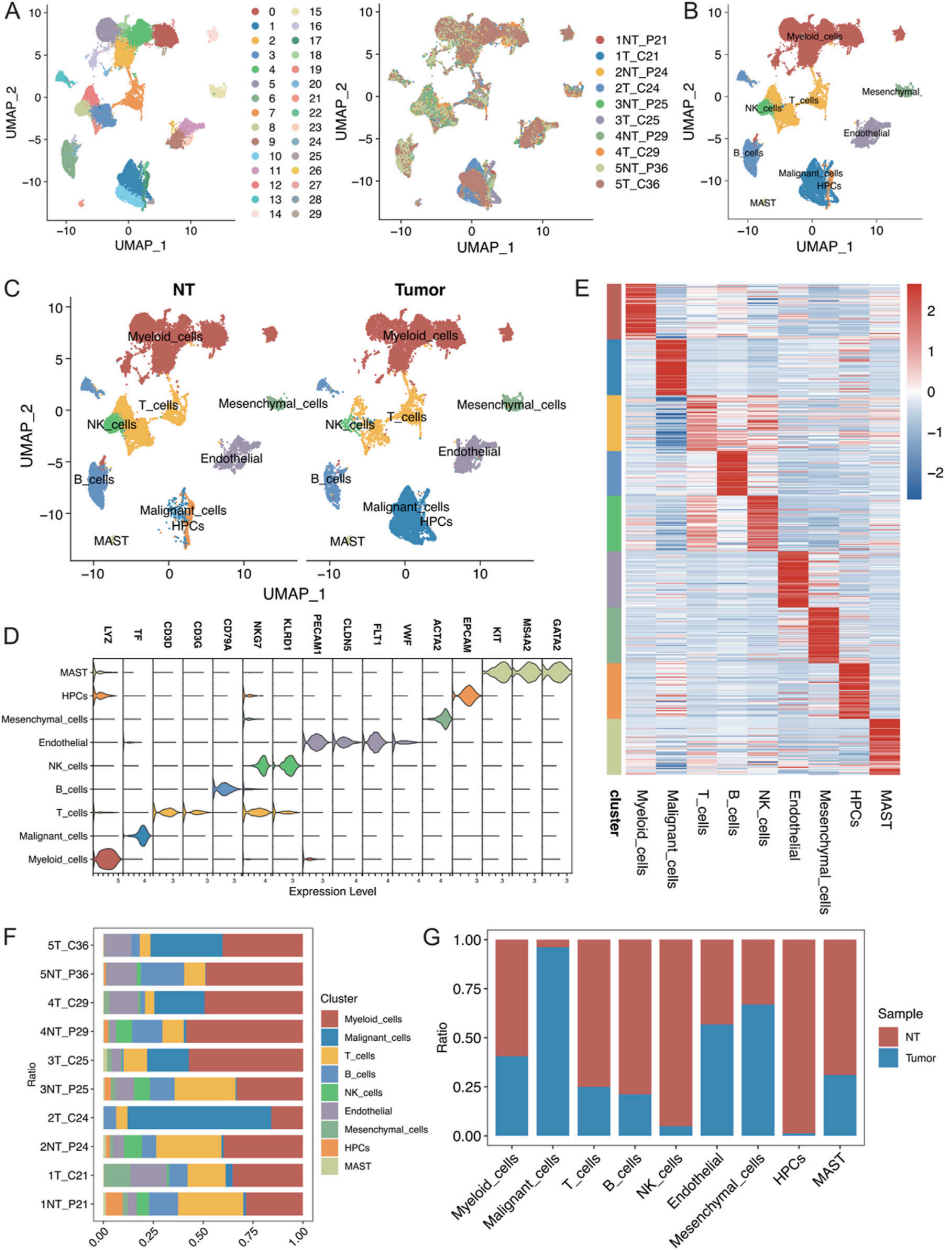
**(A)** Visualization of single-cell RNA-seq data from the GSE242889 dataset using UMAP. The left panel displays cluster subclustering, and the right panel shows the sample source for each cell. **(B)** UMAP plot illustrating the distribution of 9 cell types, with cells colour-coded according to their type. **(C)** UMAP plot depicting the distribution of cell types in the nontumor (NT) and tumour groups, with cells colour-coded according to their type. **(D)** Violin plot demonstrating the specificity of cell type annotations by marker. **(E)** Heatmap showing the specificity of cell type annotations with the names of different cell types labelled at the bottom. **(F)** Stacked bar graph displaying the percentages of different cell types in each sample. **(G)** Stacked bar graph illustrating the proportional distribution of each cell type in the NT group and tumour group.

The present study analysed 10 samples from the GSE242889 dataset, which included five patients with HCC. Among these patients, three patients exhibited MVI, while two patients did not exhibit MVI. HCC tissue and adjacent nontumor tissue were collected from each patient for single-cell RNA sequencing. After performing stringent quality control and removing duplicate cells, 42,070 cells were retained for analysis. These cells were categorized into 30 distinct clusters, and the origin of each cell was visualized (Figure 1A; Supplementary Figure S1A, B). The cell clusters were annotated using classical cell marker genes (Li et al., 2024), and they were classified them into 9 cell types, illustrating the distribution of cells in both cancerous and paracancerous samples (Figures 1B, C; Supplementary Figure S1C, D, average number of detected genes per defined cell type(Supplymentary Data.xlsx)). The following cell types were identified: myeloid cells with high LYZ expression; malignant cells with high TF expression; T cells with high CD3D and CD3G expression; B cells with high CD79A expression; NK cells with high NKG7 and KLRD1 expression; endothelial cells with high PECAM1, CLDN5, and FLT1 expression; mesenchymal cells with high ACTA2 expression; and haematopoietic progenitor cells (HPCs) with high EPCAM, KIT, MS4A2, and GATA2 expression (Figure 1D). The heatmap in Figure 1E displays the top 50 genes highly expressed in each cell type, highlighting the specificity of their compartmentalization. In this dataset, myeloid cells constituted the largest proportion of cells, followed by T cells (Supplementary Figures S1A–B). Figures 1F, G show the proportions of the cell types in different samples, in which the proportions of myeloid cells, malignant cells, and T cells were greater. Moreover, the malignant cells were almost exclusively derived from tumour tissues, demonstrating significant heterogeneity between samples.

### 3.2 Malignant tumour cells with MVI exhibit increased invasive and metastatic potential

**FIGURE 2:**
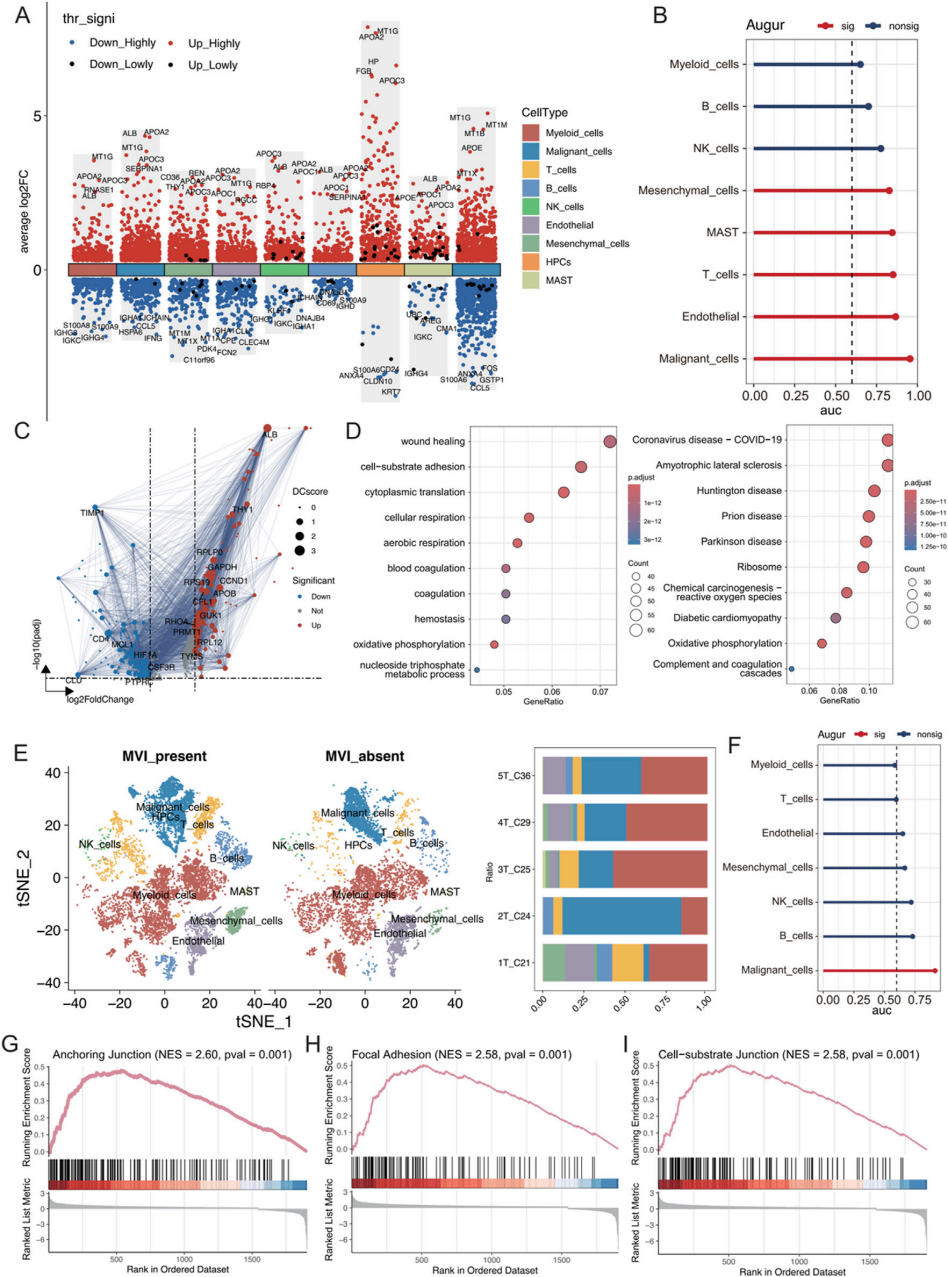
**(A)** Differential gene expression in each cell type was analysed using the Wilcoxon rank-sum test. “High” represents an adjusted p value less than 0.05, and “Low” indicates an adjusted p value less than 0.1. **(B)** AUC scores of transcriptomic perturbations were determined using the random forest framework, indicating more intense transcriptomic perturbations in cells from the NT and tumour groups. **(C)** Volcano plots showing differentially expressed genes in endothelial cells between the NT and tumour groups, with DC representing the strength of the PPI network interactions based on the STRING database. **(D)** GO and KEGG enrichment analyses of differentially expressed genes. **(E)** t-SNE visualization of each cell type in tumours with and without MVI; the right panel shows the proportion of cell types in each tumour sample. **(F)** AUC values of transcriptomic perturbations were assessed in a random forest framework, with higher AUC values indicating more significant transcriptomic perturbations occurring in cells from the tumours with and without MVI. **(G–I)** The GSEA calculations based on differentially expressed genes from the tumour groups with and without MVI are shown for relevant pathways.

The differences in the expression of various cell types were analysed between cancerous tissues and their adjacent noncancerous counterparts using the Wilcoxon rank sum test. Genes associated with lipid metabolism, such as APOA2, APOC3, APOC1, and APOE, were significantly upregulated in multiple cell types of hepatocellular carcinoma, indicating enhanced lipid metabolic activities (Figure 2A). Additionally, using the Augur algorithm in conjunction with the random forest model (Squair et al., 2021), the cell types that exhibited significant transcriptomic perturbations across different biological states were identified. Among the nine identified cell types, malignant cells showed the most significant transcriptomic changes (area under the curve (AUC) = 0.955), followed by endothelial cells (AUC = 0.866) and T cells (AUC = 0.851) (Figure 2B). In particular, the changes in endothelial cells were important. The DEGs in both states were analysed, and protein‒protein interaction (PPI) network mapping was performed using the STRING database (Figure 2C). Subsequent Gene Ontology (GO) and Kyoto Encyclopaedia of Genes and Genomes (KEGG) analyses of these genes revealed alterations in cellular connectivity and pathways related to oxidative phosphorylation (Figure 2D; Supplementary Figure S2).

The cell type distribution was compared between tumour tissues with MVI (1T_C21, 2T_C24, and 3T_C25) and those without MVI (4T_C29 and 5T_C36). There was a greater abundance of T cells, B cells, and NK cells in tumours with MVI than in those without MVI, suggesting a more active immune microenvironment in these samples (Figure 2E; Supplementary Figure S3A). Myeloid cells were the most numerous, and there was a significant preference for the distribution of different malignant cell subtypes based on MVI status (Supplementary Figures S3B–D). Furthermore, the Augur algorithm indicated that malignant cells experienced the greatest transcriptome perturbation in both MVI and non-MVI tumours (AUC = 0.914, Supplementary Figure S2F), underscoring a significantly active immune microenvironment (Supplementary Figures S2E, F). Gene set enrichment analysis (GSEA) on the differentially expressed genes in malignant cells from MVI and non-MVI tumours was performed. The anchoring junction, focal adhesion, and cell-substrate junction pathways were significantly activated in MVI-positive malignant cells, confirming their increased invasiveness and metastatic potential (Figures 2G–I). These findings highlighted the critical role of MVI in the progression of hepatocellular carcinoma and identified potential targets for future therapeutic strategies.

### 3.3 Gene expression heterogeneity of different malignant cell subtypes in microvascular invasion

**FIGURE 3:**
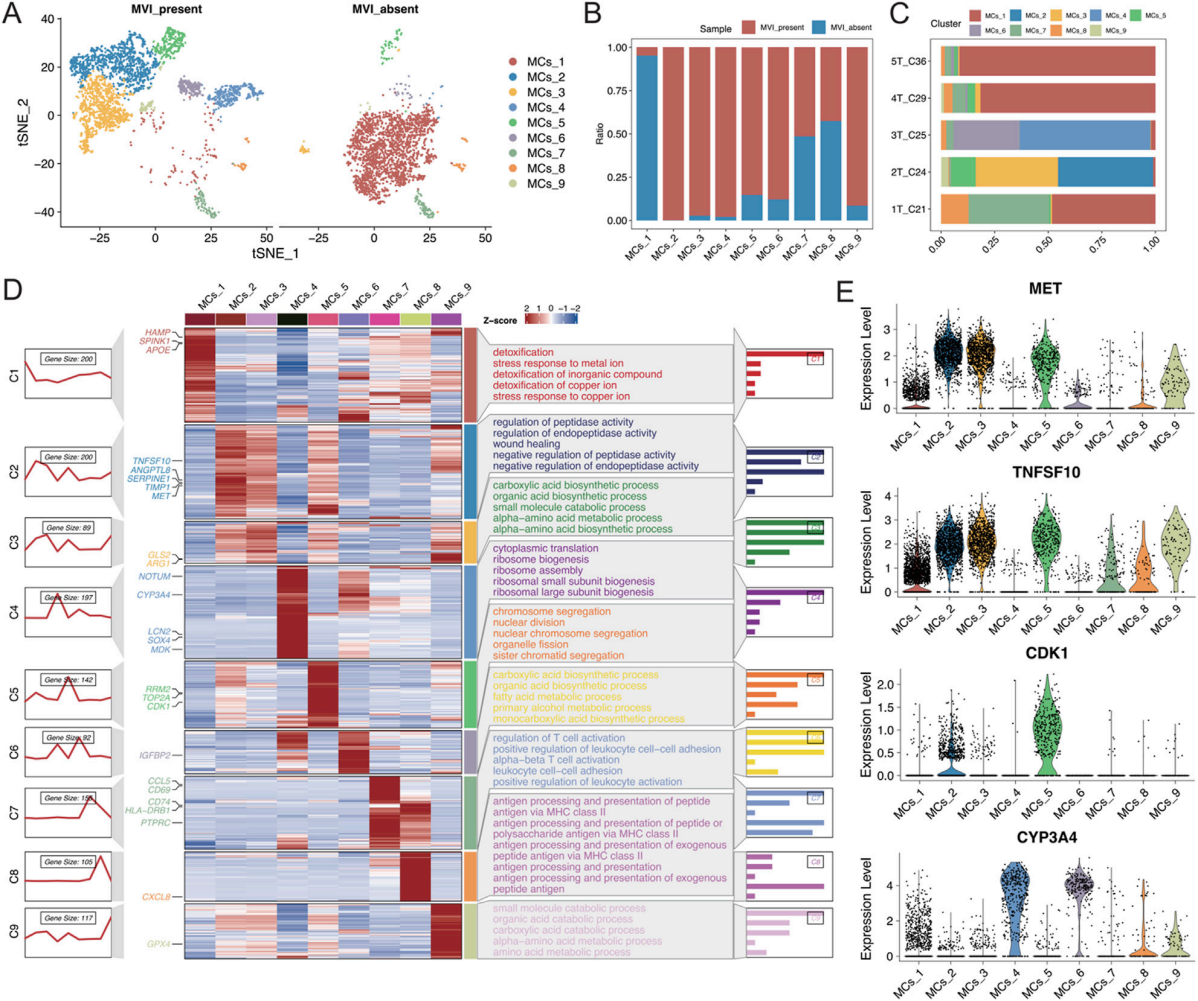
**(A)** t-SNE plot illustrating the results of malignant cell segmentation, with different colours representing various cell types. The left plot shows the group with MVI, and the right plot shows the group without MVI. **(B)** Stacked bar graph displaying the proportions of malignant cell subgroups in each group. **(C)** Stacked bar graph illustrating the proportion of malignant cell subgroups in each sample. **(D)** Left: A series of graphs illustrating the dynamic patterns of representative differentially expressed genes (DEGs) across each malignant cell population. Middle panel: Heatmap showing representative DEGs between each cell cluster. Right panel: Representative enriched Gene Ontology (GO) terms for each cluster. **(E)** Expression levels of MET, TNFSF10, CDK1, and CYP3A4 in each malignant cell subgroup.

A detailed reclassification of malignant cells in tumour tissues was performed, which identified nine distinct cell subtypes (Figure 3A). Analysis of the distribution of these subtypes in samples with and without MVI elucidated that the MCs_1 subtype was predominantly found in samples without MVI, whereas the MCs_2 to MCs_6 and MCs_9 subtypes were present in samples with MVI (Figures 3B, C). To further explore gene expression patterns, the gene expression scores were calculated for individual cells, which reflected their relative gene expression levels. These scores were used to employ an unsupervised clustering approach to visualize the unique gene expression patterns across different subtypes (Figure 3D).

The MET and TNFSF10 receptor tyrosine kinases, both highly expressed in subtype 2, were closely associated with the proliferation, survival, invasion, and metastasis of hepatocellular carcinoma cells. Aberrant activation of MET has been shown to correlate with progression and poor prognosis in hepatocellular carcinoma (Comoglio et al., 2018; Gupta et al., 2021; Huang et al., 2019). Moreover, high expression of CDK1 in subtype 5 is closely associated with the development of many cancers, including hepatocellular carcinoma (Malumbres and Barbacid, 2009). CYP3A4, which is highly expressed in subtypes 4 and 6, is the most abundant cytochrome P450 enzyme in the liver. Targeting CYP3A4 in oxidative metabolism has been reported to be an important strategy for enhancing the sensitivity of hepatocellular carcinoma cells to chemotherapeutic drugs (Figure 3E) (He et al., 2021; Özkan et al., 2021). Together, these findings revealed that each malignant cell subtype exhibits distinct functional characteristics. Subtypes with high expression of oncogenes are significantly correlated with MVI-positive samples, highlighting their potential role in the aggressive behaviour of these tumours.

### 3.4 Activation of angiogenic and mesenchymal transition pathways in malignant cell subtypes with MVI

**FIGURE 4:**
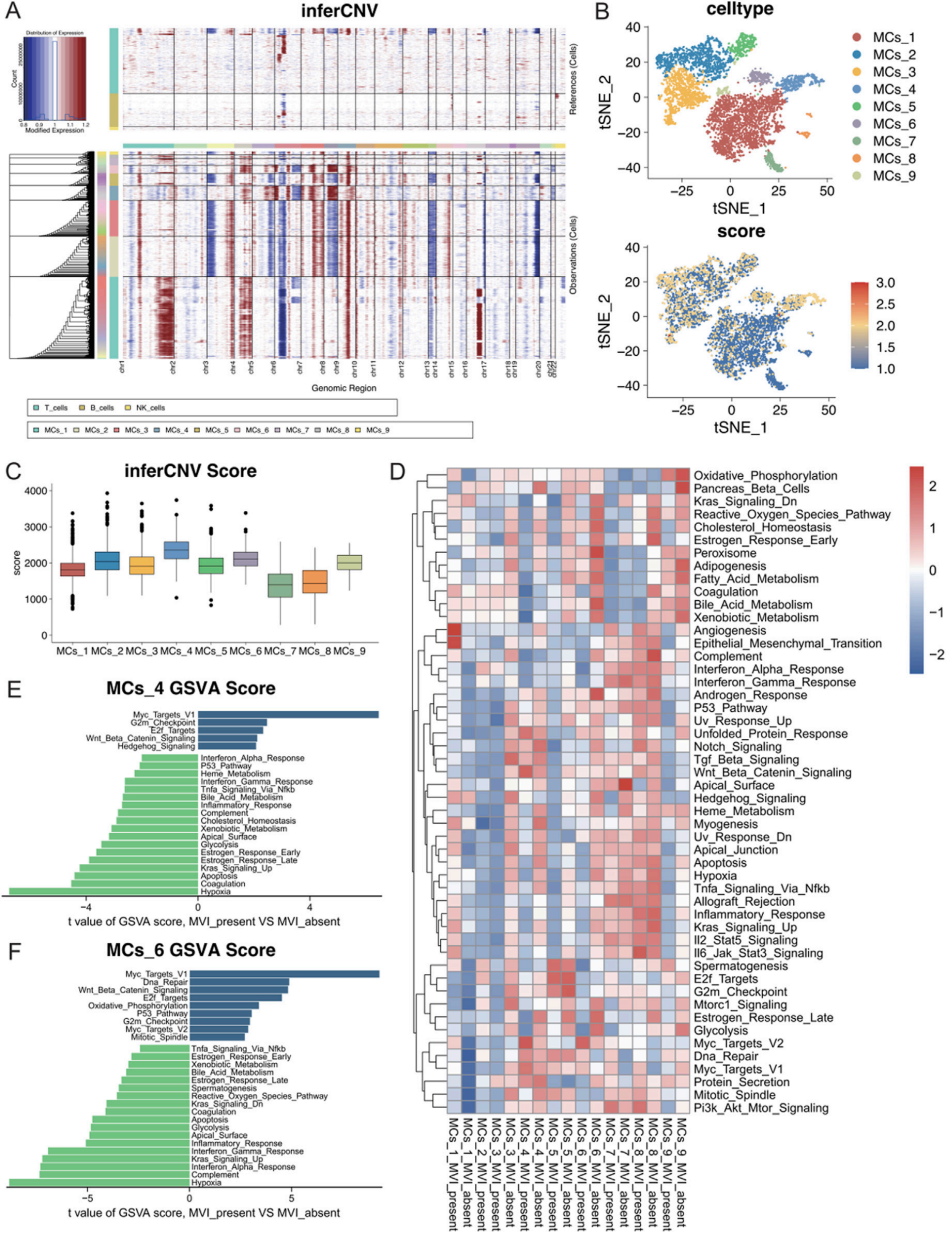
**(A)** Copy number variations in each malignant cell subtype according to the inferCNV analysis using T cells, B cells, and NK cells as references. **(B)** The CNV scores of each cell are mapped onto the malignant cell t-SNE map, with varying shades of colour representing the CNV scores. **(C)** Box line plots depicting CNV scores for nine different malignant cell subtypes. **(D)** GSEA heatmap of the 50 marker gene sets from the MSigDB database, with each subgroup of malignant cells presented according to their status in the groups with and without MVI. **(E)** Quantitative analysis of pathway differences in the MCs_4 subtype with and without MVI. **(F)** Quantitative analysis of pathway differences in the MCs_6 subtype with and without MVI.

To investigate the differences between malignant cell subtypes with MVI in hepatocellular carcinoma, copy number variation (CNV) analyses were conducted for subgroups of malignant cells with or without MVI. The inferCNV tool was used to perform a detailed assessment of intratumoral CNV heterogeneity in the subgroups. As anticipated, the CNVs exhibited significant heterogeneity among the different malignant cell subgroups (Figure 4A). Chromosomes four and nine showed significant copy number amplifications in some subgroups, whereas chromosomes 6 and 13 had significant deletions. The CNVs of each cell were visualized by a t-SNE plot (Figure 4B), and the quantitative CNV scores of each malignant cell subtype were visualized using a boxplot (Figure 4C). The CNV levels of the MCs_4 subtype were greater than those of the other subtypes, suggesting a more malignant phenotype. Similarly, the MCs_2 and MCs_6 subtypes also displayed elevated CNV levels.

Further analysis of the cancer signature gene sets (hallmark gene sets) from MSigDB was performed to assess the expression of each malignant cell subtype across different pathways (Figure 4D). The MCs_1 subtype significantly upregulated the angiogenesis and epithelial–mesenchymal transition (EMT) pathways in tumours with MVI, which indicated that a distinct gene expression pattern promoted tumour angiogenesis. In contrast, the MCs_4 and MCs_6 subtypes upregulated multiple pathways.

A differential expression analysis for MCs_4 and MCs_6 with or without MVI was conducted using limma. The Myc target pathway was significantly activated in both subtypes with MVI, suggesting rapid proliferation and metabolic reprogramming of tumour cells, which may contribute to their invasive and metastatic capacities in MVI. Moreover, the upregulation of the Wnt/Beta-Catenin and E2F Targets pathways further confirmed the high growth and invasive potential of these tumour cells (Figures 4E, F). Differences in the remaining malignant cell subpopulations are detailed in Supplementary Figure S4.

### 3.5 Single-cell trajectory analysis of MVI malignant cell subpopulations in hepatocellular carcinoma patients

**FIGURE 5:**
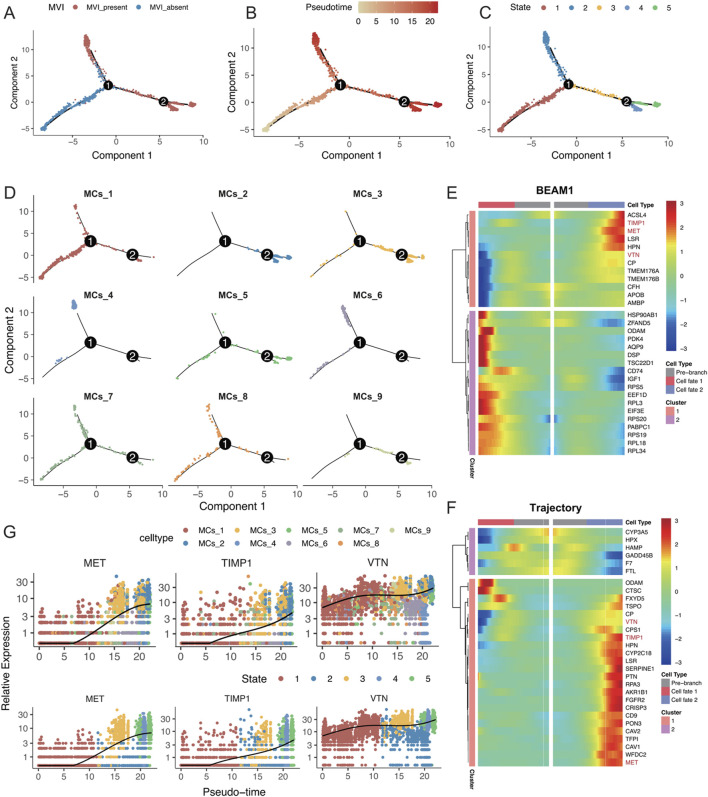
**(A–D)** Monocle analysis for inferring the trajectory of malignant cell subsets, coloured by **(A)** MVI status, **(B)** time of inference fitting, **(C)** assumed status during Monocle fitting, and **(D)** origin of the malignant cell subset. **(E)** Pseudotemporal heatmap displaying the BEAM analysis of the top 30 genes showing significant changes before and after node 1. **(F)** Pseudotemporal heatmap highlighting the top 30 genes that underwent significant changes along the trajectory. **(G)** Expression levels of MET, TIMP1, and VTN, coloured by malignant cell subgroup category and inferred developmental status as pseudotime progressed.

To analyse the changes in different malignant cell subtypes with and without MVI, the Monocle software package was used to perform a time-series analysis of the nine malignant cell subtypes, which identified five distinct cell states. The state without MVI was identified as the starting point of the trajectory, while the state with MVI marked the endpoints of two differentiation trajectories (Figures 5A–C). The MCs_4 and MCs_6 cell subtypes predominantly focused on one of the trajectory endpoints, whereas the MCs_2 and MCs_3 cell subtypes focused on the other, suggesting that these subtypes may represent two distinct fates (Figure 5D). Further analysis explored changes in gene differentiation trajectories before and after node 1, and the top 30 and top 200 genes were identified (Figure 4E; Supplementary Figure S5A). By tracking the dynamic changes in the expression of genes along the trajectories (Figure 4F), two distinct expression patterns among the top 30 genes were identified, as determined by two statistical approaches for identifying DEGs. This finding was partially validated by BEAM analyses at node 2 (Supplementary Figures S5B, C).

Intersection analysis of these top 30 genes identified MET, TIMP1, and VTN as key genes for progression along the trajectory, with consistent findings using both statistical methods. These genes were highly expressed in the MCs_4, MCs_5, and MCs_6 cell subtypes, as well as in states four and 5 (Figure 4G; Supplementary Figure S5D). Research has shown that MET activates signalling pathways, such as the PI3K/AKT and RAS/MAPK pathways, promoting the proliferation, survival, and migration of hepatocellular carcinoma cells, as well as influencing vascular endothelial cells to promote neointimal formation (Bussolino et al., 1992; Bladt et al., 1995; Huh et al., 2004). TIMP1 regulates the remodelling of the extracellular matrix by inhibiting metalloproteinase activity, which helps tumour cells adapt to changes in the matrix, thereby enhancing cell survival by inhibiting apoptosis (Guccini et al., 2021; Justo and Jasiulionis, 2021), potentially affecting blood vessel formation during MVI. Vitronectin (VTN) promotes tumour cell migration and invasion by enhancing adhesion to the stroma and interactions with cell surface integrins, possibly supporting tumour nutrient and oxygen supply by influencing vascular endothelial function and promoting neointimal formation (Wei et al., 2018; Zhu et al., 2015). In conclusion, the present study highlighted the critical roles of MET, TIMP1, and VTN in the progression of MVI in hepatocellular carcinoma, underscoring their previously unreported contributions to this process (Figures 5E–F).

### 3.6 Immune cell dynamics and MVI-dependent alterations in HCC microenvironment

**FIGURE 6:**
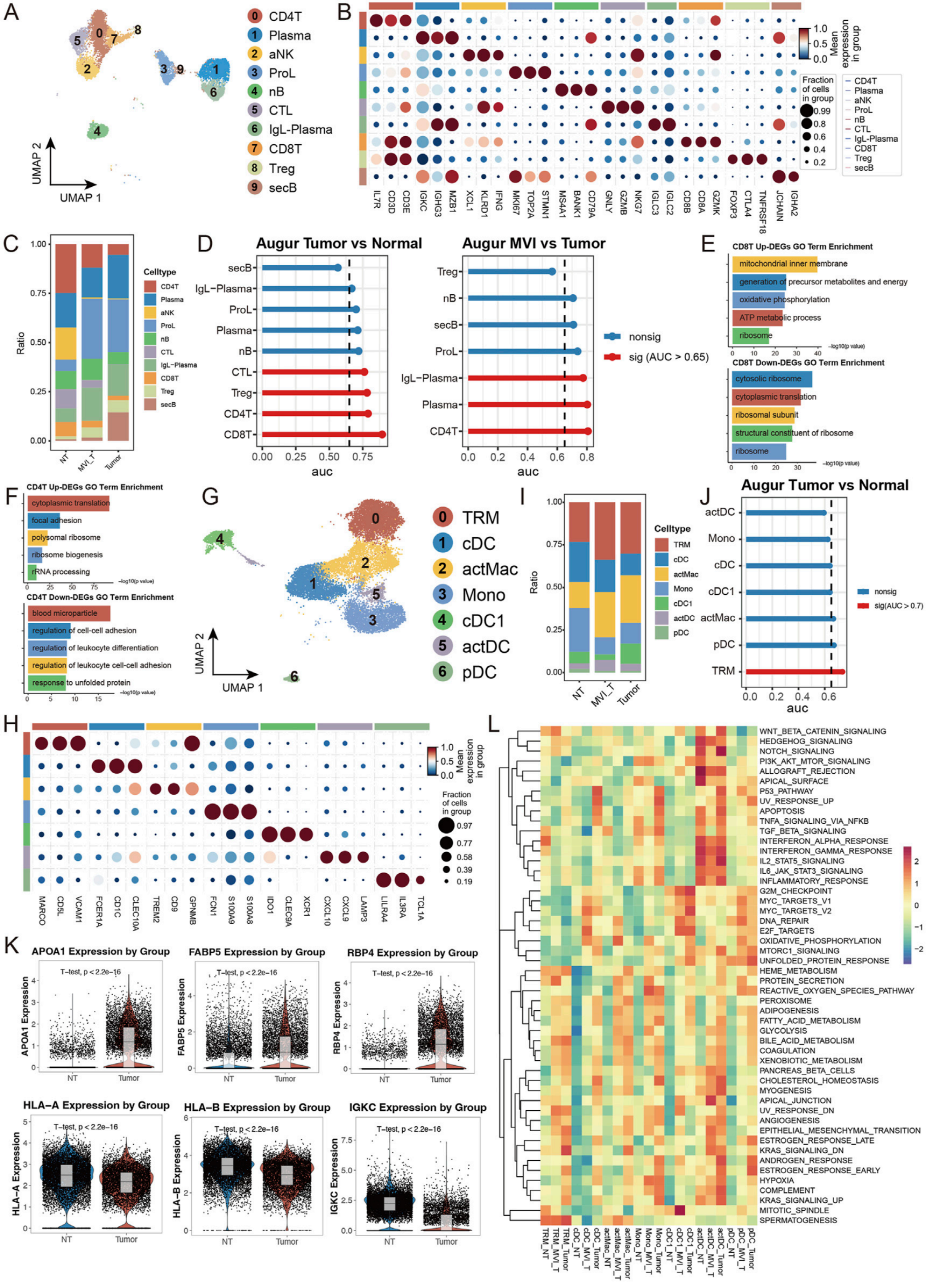
Single-cell Transcriptomic Analysis Reveals Heterogeneous Characteristics of Lymphoid and Myeloid Cells: **(A)** UMAP dimensionality reduction plot of lymphoid cell subpopulations, identifying ten major subtypes: CD4^+^ T cells (CD4T), Plasma cells, Activated NK cells (aNK), Proliferating lymphocytes (ProL), Naive B cells (nB), Cytotoxic lymphocytes (CTL), IgL + Plasma cells (IgL-PC), CD8^+^ T cells (CD8T), Regulatory T cells (Treg), and Secretory B cells (secB). **(B)** Expression profiles of characteristic marker genes for each lymphoid cell subpopulation, used for cell identity confirmation. **(C)** Stacked bar plot showing the proportion distribution of lymphoid cell subtypes in tumor and normal groups. **(D)** Transcriptional perturbation analysis using Augur. **(E, F)** Gene set enrichment analysis of differentially expressed genes in CD8T cells comparing Tumor vs. Normal, separately analyzed for **(E)** upregulated and **(F)** downregulated genes. **(G)** UMAP dimensionality reduction plot of myeloid cell subpopulations, identifying seven major subtypes: tissue-resident macrophages (TRM), Conventional Dendritic Cells (cDC), Activated Macrophages (actMac), Monocytes (Mono), Type 1 cDC (cDC1), Activated DC (actDC), and Plasmacytoid DC (pDC). **(H)** Expression profiles of characteristic marker genes for each myeloid cell subpopulation. **(I)** Stacked bar plot showing the proportion distribution of myeloid cell subtypes in tumor and normal groups. **(J)** Transcriptional perturbation analysis using Augur. **(K)** Violin plots showing representative gene expression. **(L)** Heatmap of GSEA enrichment analysis based on 50 characteristic gene sets from MSigDB, displaying functional features of myeloid cell subpopulations across different groups.

To comprehensively understand the alterations in the tumor immune microenvironment, we conducted a systematic analysis of lymphoid and myeloid cells in our dataset. Using unsupervised clustering methods, we initially classified lymphoid cells into 10 distinct subpopulations and annotated their cell types based on subgroup-specific highly expressed genes (Figures 6A, B). Cell abundance analysis revealed significant enrichment of proliferating lymphocytes (ProL) and IgL-expressing plasma cells (IgL-Plasma) in the tumor group, while the proportion of CD4^+^ T cells (CD4T) was notably decreased. Interestingly, in tumors positive for microvascular invasion (MVI), secretory B cells (secB) were significantly reduced compared to MVI-negative tumors, whereas CD4T showed an opposite trend, indicating a close relationship between MVI status and immune cell composition (Figure 6C). Transcriptomic analysis demonstrated that CD8^+^ T cells (CD8T) exhibited the most significant transcriptional changes in tumor *versus* normal tissue comparisons, while CD4T showed the most prominent alterations in MVI-related comparisons (Figure 6D). Detailed analysis revealed that genes related to oxidative phosphorylation and energy transfer were significantly upregulated in tumor CD8T, while cytoplasmic ribosome-related pathway genes were downregulated, suggesting metabolic reprogramming and functional state changes (Figure 6E). In MVI-positive samples, upregulated genes in CD4T were primarily enriched in cytoplasmic translation and focal adhesion pathways, indicating a potentially highly activated state (Figure 6F).

In the myeloid cell analysis, we identified seven distinct subpopulations and performed detailed analysis of their functional marker expression profiles (Figures 6G, H). The tumor group showed decreased proportion of monocytes (Mono) but increased proportions of tissue-resident macrophages (TRM) and activated macrophages (actMac) (Figure 6I). Notably, in MVI-positive tumors, the proportion of type 1 conventional dendritic cells (cDC1) was significantly lower than in MVI-negative tumors (Supplementary Figure S6I). Transcriptomic analysis indicated that TRM underwent the most significant transcriptional remodeling. Specifically, in tumor TRM, genes related to lipid metabolism and transport (such as APOA1) and nutrient metabolism and transport (such as RBP4, FABP5) were significantly upregulated, suggesting these cells may have acquired special metabolic functions and participate in nutrient redistribution within the tumor microenvironment. Meanwhile, the downregulation of antigen presentation molecules (HLA-A/B) and immunoglobulin (IGKC) suggested potentially suppressed immune functions, facilitating tumor immune escape (Figures 6J, K). Assessment of cell subpopulation functional states using MSigDB gene sets revealed that myeloid cell subpopulations in normal tissue generally exhibited low pathway activity levels, while differences in MVI status were reflected in the activation levels of multiple functional pathways, further supporting the association between microvascular invasion and immune cell functional changes (Figure 6L). These findings reveal the dynamic characteristics of immune cells in the hepatocellular carcinoma microenvironment and highlight the crucial role of microvascular invasion in reshaping the tumor immune microenvironment, providing new insights for understanding HCC progression mechanisms and developing immunotherapy strategies.

### 3.7 Upregulated laminin and VEGF signalling pathways in the MVI malignant subgroups

**FIGURE 7:**
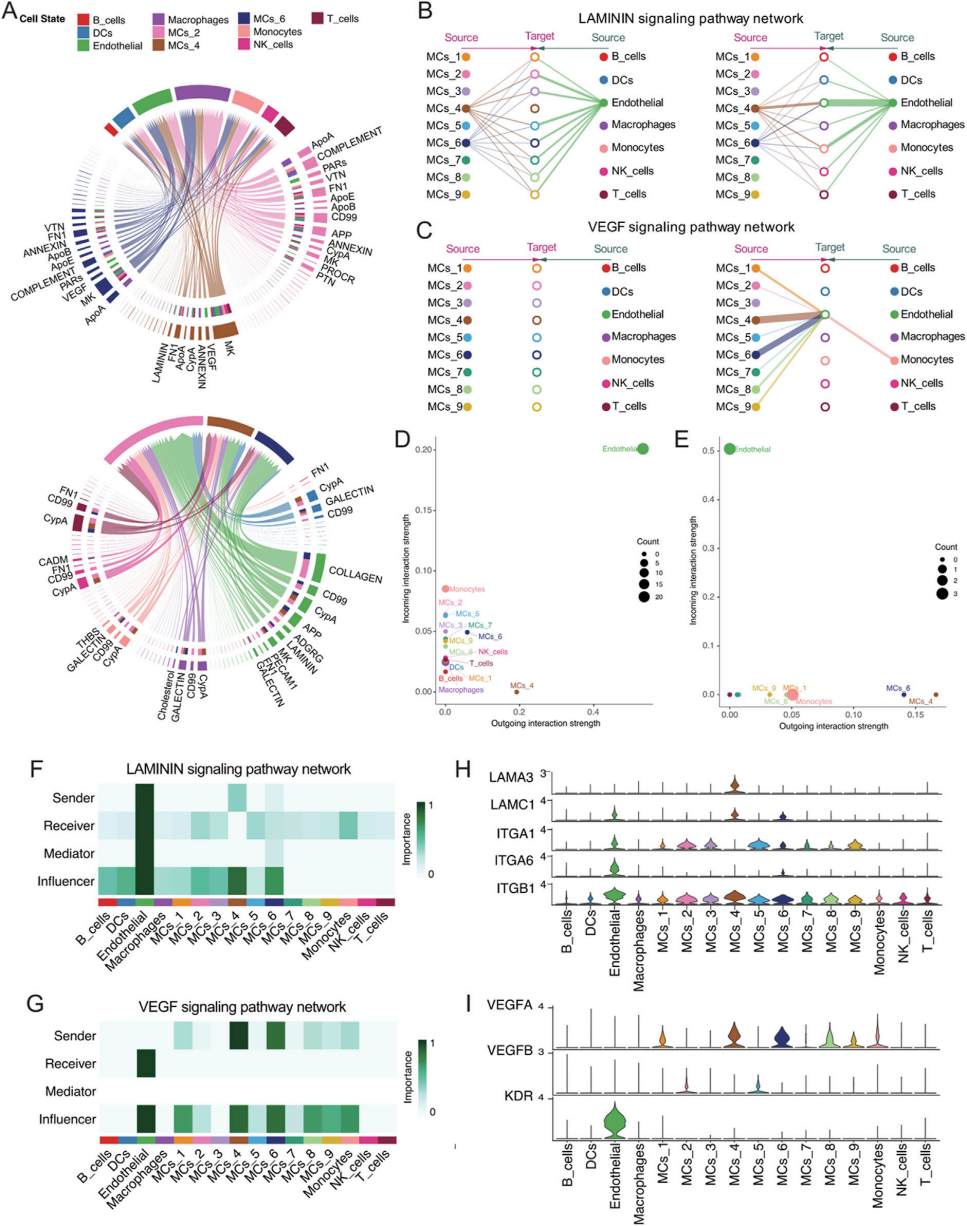
**(A)** Line graphs demonstrating receptor‒ligand interactions involving the MCs_2, MCs_4, and MCs_6 subtypes as both signal senders and receivers. **(B, C)** Hierarchical diagrams depicting inferred intercellular communication networks for **(B)** laminin signalling and **(C)** VEGF signalling. Interactions are categorized into sources and targets, marked with solid and hollow circles, respectively, with the width of the connecting lines indicating the probability of communication. **(D, E)** Dot plots showing the **(D)** senders and receivers of the laminin signalling pathway, as well as the **(E)** senders and receivers of the VEGF signalling pathway. The X- and Y-axes represent the total outgoing and incoming communication probabilities associated with each group, respectively. The size of the dots correlates positively with the number of inferred links (efferent and afferent) associated with each cell block, and the colours of the dots represent different cell groups. **(F, G)** Heatmaps showing network centrality scores for **(F)** laminin signalling and **(G)** VEGF signalling. **(H, I)** Violin plots illustrating the expression of molecules involved in **(H)** laminin signalling and **(I)** VEGF signalling across all cell types.

The three malignant cell subpopulations with high CNV, namely, MCs_2, MCs_4, and MCs_6, are potentially associated with the progression of MVI in hepatocellular carcinoma. To further explore the communication between these malignant cells and other cell types, the myeloid cells that exhibited the highest percentage of interaction were subdivided (Supplementary Figures S5E, F) and subjected to CellChat analysis (Supplementary Figure S6A). Significant intercellular communication was identified within all three malignant cell subpopulations (Supplementary Figures S6B–E). Notably, the MCs_2 subtype demonstrated the most extensive receptor‒ligand interactions among the three subtypes, engaging both the ApoA and complement signalling pathways, as well as acting as both a receiver and a sender of signals. The VTN, CypA, and VEGF signalling pathways were significantly activated in these subpopulations, underscoring the enhanced invasiveness and angiogenic capacity of these tumour cells (Figure 7A). Among the receptor‒ligand interactions, the laminin and VEGF signalling pathways were the most significantly activated, suggesting remodelling of the tumour microenvironment and the extracellular matrix.

By binding to cell surface integrins, laminin activates multiple downstream pathways that significantly influence cell attachment, migration, and survival. This mechanism is crucial for maintaining tissue architecture and promoting tumour invasiveness and metastasis (Rick et al., 2019). Single-cell data analysis revealed that two malignant cell subpopulations, namely, MCs_4 and MCs_6, were the primary emitters of laminin signalling, with the MCs_4 subtype delivering stronger signals, particularly to the endothelial cell population (Figure 7B). The MCs_4 subtype was the dominant signaller of endothelial cells, and all three malignant cell subpopulations were identified as key influencers of this pathway (Figures 7D, F). The expression of laminin signalling-related molecules is depicted in Figure 7H. Moreover, the VEGF signalling pathway, which is known to support tumour growth and metastasis by promoting angiogenesis through vascular endothelial cell proliferation, migration, and neointima formation (Carmeliet, 2005), was also highlighted. The present study demonstrated that the endothelial cells were the primary receivers of VEGF signalling, with the MCs_4 and MCs_6 subtypes acting as the main emitters (Figures 7C, E). All three malignant cell subpopulations were identified as potential effectors of VEGF signalling (Figure 7G). Analysis of the expression of molecules in the VEGF pathway (Figure 7I) revealed that VEGFA was highly expressed in the MCs_4 and MCs_6 subtypes, whereas VEGFB was predominantly expressed in the MCs_2 subtype.

The patterns of afferent and efferent signals across all malignant cell subpopulations were analysed using cophenetic- and silhouette-based methods (Supplementary Figures S6F, G). In all malignant cell subgroups, the MCs_5 subtype emerged as the most significant signalling efferent, followed by the MCs_2 and MCs_4 subtypes. Conversely, the MCs_2 subtype was the most significant signalling afferent, followed by the MCs_3 and MCs_5 subtypes. The MCs_2, MCs_4, and MCs_6 subtypes displayed similar afferent and efferent patterns, suggesting a potential association (Supplementary Figures S7B–D).

### 3.8 MVI malignant cell subpopulation-related traits predict patient survival

**FIGURE 8:**
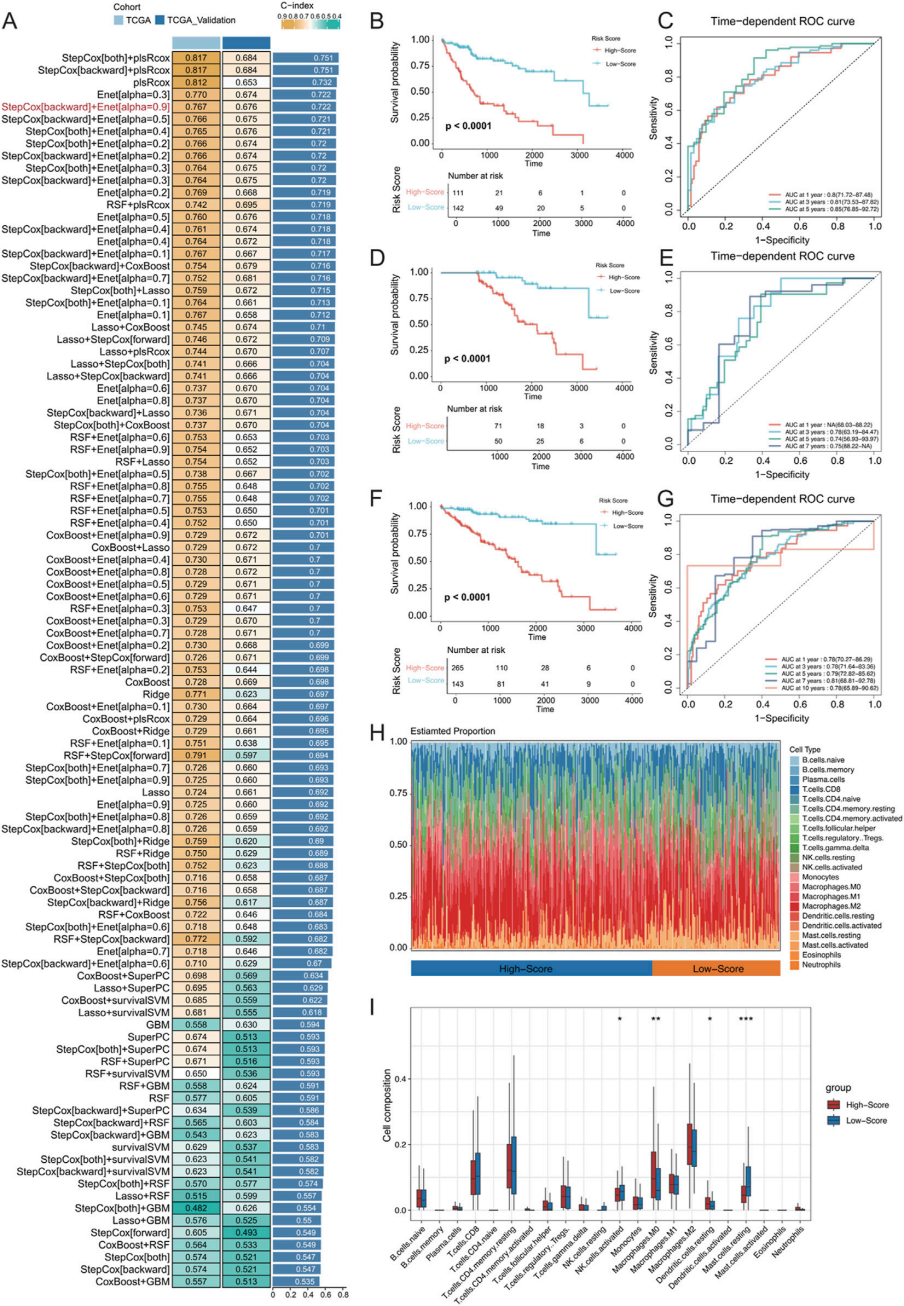
**(A)** Development and validation of the consensus characterization MRS model through an integrated machine learning-based procedure involving a total of 101 prognostic models using the LOOCV framework. The C-index of each model was calculated for all training and validation datasets. **(B)** Kaplan‒Meier (K–M) survival curves of the MRS prognostic models in the training set. **(C)** ROC curves for the training set, evaluating the performance of the models using 1-year, 3-year, and 5-year AUC values. **(D, E)** K‒M survival curves and ROC curves for the MRS prognostic models in the validation set. **(F, G)** K‒M survival curves and ROC curves for the MRS prognostic model across all samples. **(H)** Identification of immune infiltration in the high- and low-risk groups differentiated by the model using the CIBERSORT approach. The box plot shows the immune cell infiltration for each type of immune cell. **(I)** * represents *p* < 0.05, ** represents *p* < 0.01, and *** represents *p* < 0.001.

Data from TCGA-LIHC were utilized to fit matched prognostic data, and the data were screened for genes associated with patient prognosis using one-way Cox regression, which resulted in 673 genes (p < 0.0001). The genes specifically expressed in the MCs_2, MCs_4, and MCs_6 subtypes were intersected with those obtained by one-way Cox regression, which resulted in 452 genes. These models were fitted into 101 predictive models within the LOOCV framework. TCGA-HCC dataset was divided into training and validation sets at a 1:1 ratio, and the C-index was calculated for each model in both sets. The model combination of StepCox[both] + plsRcox yielded the highest average C-index (0.751), utilizing 174 features for survival prediction. However, the StepCox[backward] + Enet[alpha = 0.9] strategy, using only 27 features, achieved an average C-index of 0.722 (Figure 8A). Subsequently, an MVI malignant cell-related feature (MRS) model was constructed using the latter strategy.

In both the training and validation sets of TCGA-LIHC cohort, the low-risk group exhibited significantly longer OS (Figures 8B, D; OS, p < 0.0001). In the training set, the MRS model determined AUC values of 0.8 (71.72–87.48), 0.81 (73.53–87.82), and 0.85 (76.85–92.72) for 1-, 3-, and 5-year OS, respectively. Due to sample size limitations, the 1-year AUC was not calculated in the validation set, but the AUC values for 3-, 5-, and 7-year OS were 0.78 (63.19–84.47), 0.74 (56.93–93.97), and 0.75 (88.22-NA), respectively (Figures 8C, E). These results demonstrated that the MRS model, which is based on MVI malignant cell subtypes, is an effective predictive tool with satisfactory specificity and sensitivity. When combining the training and validation sets, the low-risk group consistently had significantly longer OS than the high-risk group across all samples (Figure 8F), with AUC values of 0.78 (70.27–86.29), 0.78 (71.64–83.36), and 0.79 (72.82–85.62) for at 1, 3, and 5 years, respectively (Figure 8G). Additionally, the CIBERSORT algorithm was used to analyse the proportion of infiltrating immune cells and the correlation between high- and low-risk samples in TCGA-HCC dataset (Figures 8H, I). There was a significantly greater proportion of infiltrated M0 macrophages in the high-risk group (*P* < 0.01) and a significantly lower proportion of mast cells in the resting state (*P* < 0.001).

### 3.9 MARCKSL1 promotes MVI progression through PTN-Related networks

**FIGURE 9:**
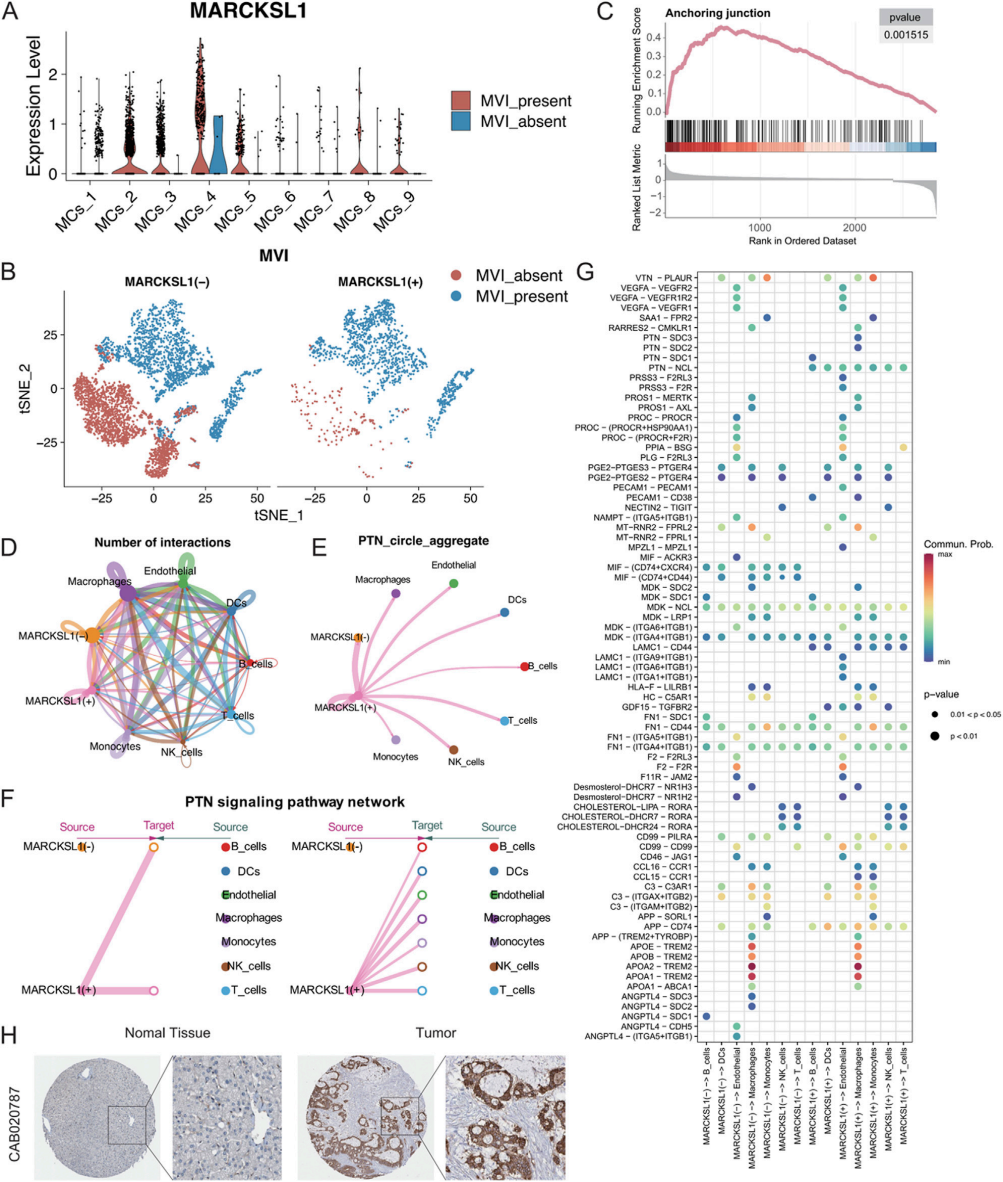
**(A)** MARCKSL1 expression across various malignant cell subgroups. **(B)** t-SNE plot displaying malignant cells coloured according to MARCKSL1 expression, where MARCKSL1 (+) represents cells expressing MARCKSL1 and MARCKSL1 (−) represents cells lacking MARCKSL1 expression. **(C)** GSEA plot indicating activation of the anchoring junction pathway in the MARCKSL1(+) group. **(D)** Circular plots illustrating the number of interactions between the indicated MARCKSL1 subgroups and immune cells. **(E)** Circular plots of the intensity of interactions of all cells involved in the PTN signalling pathway. **(F)** Hierarchical plot displaying the interaction intensity of PTN signalling pathway components with other cell types in MARCKSL1(+) cells. **(G)** Illustration of receptor‒ligand interactions generated by the MARCKSL1(+) and MARCKSL1(−) malignant cell signal senders. **(H)** Based on the Human Protein Atlas database, increased expression of MARCKSL1 was found in hepatocellular carcinoma tissue compared to normal tissue.

The 27 signature genes in the MRS model were analysed, which identified MARCKSL1, a gene involved in various cellular processes, including cell migration, cytoarchitectural adjustments, and signalling. The role of MARCKSL1 has not been extensively demonstrated in hepatocellular carcinoma (Zhao et al., 2023; Chen et al., 2021; Yadav et al., 2024). Based on our preliminary findings, we hypothesized that MARCKSL1 plays a significant role in MVI and tumour progression in hepatocellular carcinoma. MARCKSL1 was primarily expressed in the MCs_2, MCs_4, and MCs_5 subtypes, indicating the potential association with MVI and cancer malignancy (Figure 9A). MARCKSL1-positive (MARCKSL1(+)) expression was almost exclusively found in tumours with MVI, while MARCKSL1-negative (MARCKSL1(−)) expression lacked this specificity (Figure 9B). Differential gene analysis between MARCKSL1(+) and MARCKSL1(−) malignant cells was performed, and the identified genes, along with differential ploidy, were used to conduct GSEA. This analysis revealed a significant increase in the number of anchoring junction entries in MARCKSL1(+) cells (NES = 2.64, p value = 0.001) (Figure 9C).

To further explore the interactions between malignant MARCKSL1(+) and MARCKSL1(−) cells and immune cells, CellChat tools were used to analyse all receptor‒ligand pairs, which indicated that MARCKSL1(+) cells delivered significant PTN signals to other cells (Figures 9D–F). PTN-related signals are known to promote angiogenesis and extracellular matrix remodelling through MMPs (Papadimitriou et al., 2022; Perez-Pinera et al., 2008; Mentlein, 2007). The present study demonstrated that MARCKSL1(+) cells were involved in other signalling pathways, including LAMC1 and PRSS3 signalling pathways, distinguishing these cells from MARCKSL1(−) cells (Figure 9G). Thus, these findings suggested that MARCKSL1 influences the progression of MVI and cancer malignancy through PTN-related signalling, which is supported by immunohistochemistry results from the Human Protein Atlas showing greater expression of MARCKSL1 in cancerous tissues than in adjacent noncancerous tissues (Figure 9H).

**FIGURE 10:**
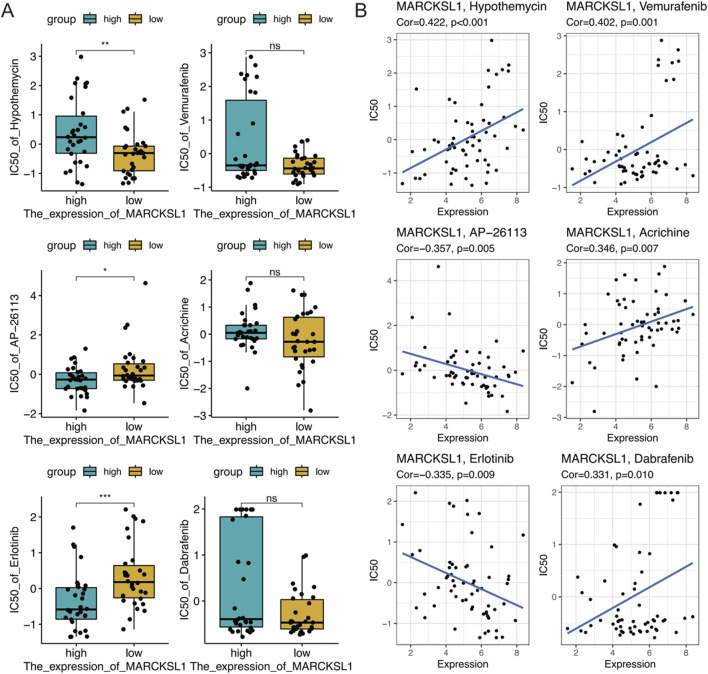
**(A)** Boxplot illustrating the gene expression and sensitivity to six drugs obtained from the NCI-60 database based on median MARCKSL1 grouping. **(B)** Investigation of the correlation between the MARCKSL1 expression level in tumour cells and drug sensitivity to six compounds.

To further explore the therapeutic value of MARCKSL1 in cancer treatment, data from drug sensitivity analyses were collected using the NCI-60 cell line panel and RNA sequencing data from cell lines in the National Cancer Institute (NCI) database. In total, 75 clinical trials and 188 FDA-approved drugs were reviewed to investigate the relationship between MARCKSL1 expression levels and the IC50s of these drugs by calculating Pearson’s correlation coefficients (Figures 10A, B). The abundance of MARCKSL1 was correlated with tumour cell resistance to several drugs, including hypothemycin, vemurafenib, AP-26113, acrichine, erlotinib, and dabrafenib.

## 4 Discussion

The present study demonstrated significant differences in the transcriptomes of malignant cells associated with the presence and absence of MVI, particularly in terms of cell adhesion capacity and enhanced tumour invasion and metastasis. While previous research focused on the multicellular ecosystem and the roles of immune and stromal cells in MVI formation (Li et al., 2024), our study investigated the heterogeneity of MVI-associated malignant cells, revealing key molecular mechanisms. Specifically, we identified the upregulation of cell junction-related pathways (e.g., cell junction, NES = 2.32, P value = 0.001; structural molecule activity, NES = 2.49, P value = 0.001) and key signaling pathways such as VEGF and laminin signaling, which may promote tumour cell invasion along vessel walls (Leong et al., 2022; Shenoy and Lu, 2016). These findings suggest that malignant cells at the MVI stage may alter their cytoskeleton and adhesion patches to more efficiently cross the extracellular matrix and vessel walls. Additionally, we identified a subpopulation of malignant cells (MCs_1) in non-MVI hepatocellular carcinoma that exhibited activation of detoxification-related pathways (e.g., detoxification of inorganic compounds and copper ions), potentially enhancing resistance to chemotherapeutic agents (Lee et al., 2012). Overall, our study complements the multicellular ecosystem perspective by providing insights into the intrinsic mechanisms of malignant cells, offering a more comprehensive understanding of MVI complexity.

According to the inferCNV results, the MCs_4 and MCs_6 subtypes were the two most malignant cell subtypes. Comparison of the pathway changes in the MCs_4 and MCs_6 subtypes in two different biological states, namely, presence of MVI and absence of MVI, indicated that the Myc target pathway was significantly activated in both subtypes in the absence of MVI. Activation of Myc has been reported to promote the metabolic activity and proliferation of endothelial cells by enhancing their glycolysis and mitochondrial function, thereby effectively promoting vascular growth and expansion (Wilhelm et al., 2016). The present findings highlighted the impact of Myc-related networks on MVI. Moreover, significant enhancements in the G2M checkpoint and E2F target pathways were also found in two subgroups of malignant cells in MVI-present tumours compared to those in MVI-negative tumours. The G2/M checkpoint is involved in cell cycle control, whereas E2F is a key element in the regulation of the cell cycle and is associated with DNA replication and cell division. The upregulation of these pathways may be the molecular basis for the rapid proliferation and invasiveness of MVI tumour cells. Additionally, the present findings indicated downregulation of the apoptosis, coagulation, and hypoxia pathways in MVI-present tumours, suggesting that malignant MVI tumour cells may promote their survival and proliferation by inhibiting natural cell death mechanisms, adjusting interactions with the microenvironment, and adapting to hypoxic conditions. These findings offer new insights into how malignant MVI cells adapt and promote tumour growth, invasion, and metastasis by regulating key biological pathways.

In the present study, malignant cells were classified based on MARCKSL1 expression into the MARCKSL1(+) and MARCKSL1(−) cells. In addition to affecting the PTN signalling pathway, MARCKSL1(+) cells exhibited enrichment of GDF15-TGFBR2 in immune cells, a feature that distinguishes them from MARCKSL1(−) cells. TGF-β is known to play a critical role in early embryonic development and adult homeostasis *in vivo* (Xu et al., 2018). In cancer, overexpression of TGF-β is closely associated with metabolic disorders, dysfunction, epithelial–mesenchymal transition, immune deficits, and cancer progression (Su et al., 2020; Liu et al., 2021). Xu and colleagues reported that the TGF-β-associated pathway, through the regulation of FOXC1, affects tumour EMT, thereby promoting MVI (Xu et al., 2012). These findings suggested that the TGF-β signalling pathway is one of the mechanisms through which MARCKSL1 promotes MVI and the malignant progression of hepatocellular carcinoma, a connection that has not yet been reported and warrants further investigation. Additionally, the interaction of LAMC1 (laminin C1) with integrin subunits (e.g., ITGA9, ITGB1, ITGA6, and ITGA1) involves extracellular matrix interactions and cell adhesion. These interactions are also specific to MARCKSL1(+), which functions as a receptor‒ligand pair and has been reported to be critical for tumour cell migration, particularly in interactions with vascular endothelial cells. These findings suggested that this pathway is also involved in the regulation of MVI by MARCKSL1.

The present study utilized single-cell analysis techniques to investigate the functional switch of malignant cells in the progression of MVI in hepatocellular carcinoma and its impact on the immune microenvironment. For the first time, the present study reported and highlighted the potential impact of the laminin- and VEGF-related pathways on MVI. MARCKSL1, a molecule not yet widely reported, was identified to be associated with PTN-related signalling pathways. High expression of this gene was closely associated with poor patient prognosis, suggesting that it may become an important prognostic marker and therapeutic target.

## 5 Limitations of the study

The present study had several limitations. First, the single-cell and prognostic data were obtained from public databases and involved a limited number of patient samples; therefore, the present findings need to be validated in larger clinical samples. Second, the present study did not include *in vitro* or *in vivo* experimental validation. Future studies will confirm the present results with additional experiments and further investigate the role of MARCKSL1 in the progression of MVI in hepatocellular carcinoma.

## Data Availability

The original contributions presented in the study are included in the article/Supplementary Materials, further inquiries can be directed to the corresponding author.
