# Supplementary material for: Single-cell sequencing reveals cell heterogeneity and aberrantly activated pathways associated with microvascular invasion in hepatocellular carcinoma
Source: Front Cell Dev Biol. 2025 Jan 29;13:1449624. doi: 10.3389/fcell.2025.1449624 (PMC11814199; doi:10.3389/fcell.2025.1449624)
Supplement: Supplementary file 1 [file DataSheet1.docx]

Supplementary Material

# Supplementary Figures


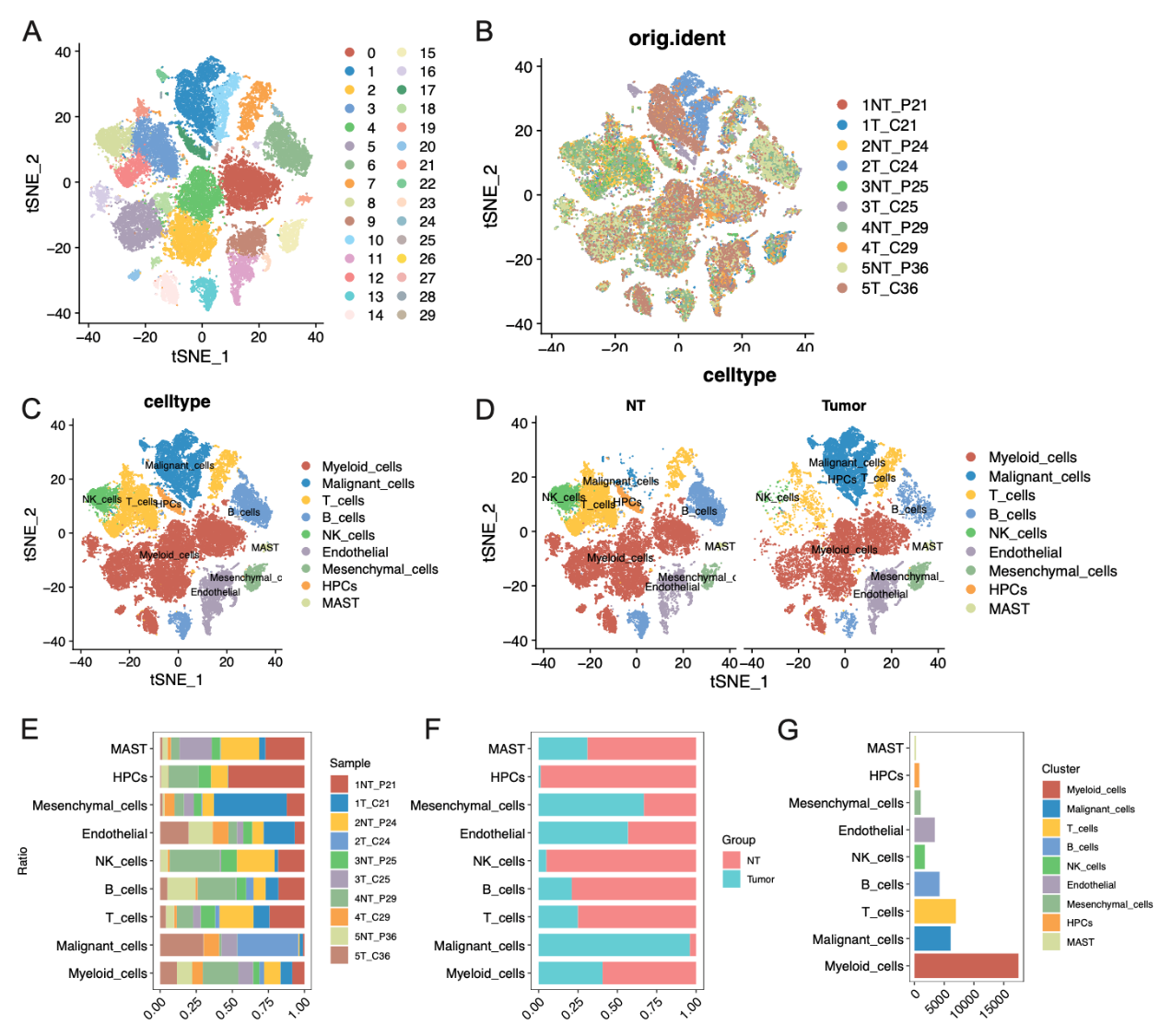


**Supplementary Figure S1:** (A) t-SNE plot illustrating the clusters calculated for individual cells. (B) t-SNE plot showing the sample source of individual cells. (C) t-SNE plot depicting the distribution of the 9 cell types, with cells colored according to type. (D) t-SNE visualization of each cell type in the NT group and Tumor group. (E-G) Stacked bar charts illustrating the: (E) sample share, (F) group share, and (G) absolute number of each cell type.


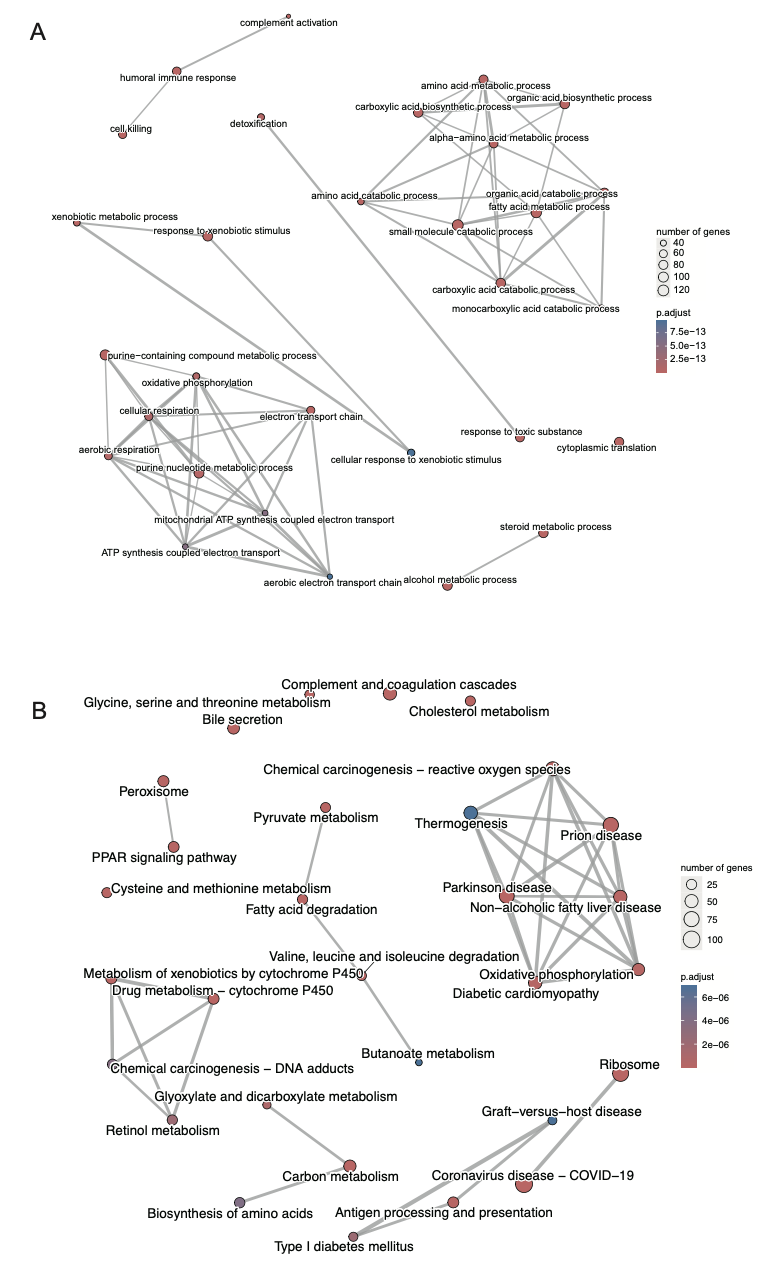


**Supplementary Figure S2:** (A-B) Network diagrams showing: (A) GO and (B) KEGG pathway enrichment analyses of endothelial cell differential genes in the T and NT groups.


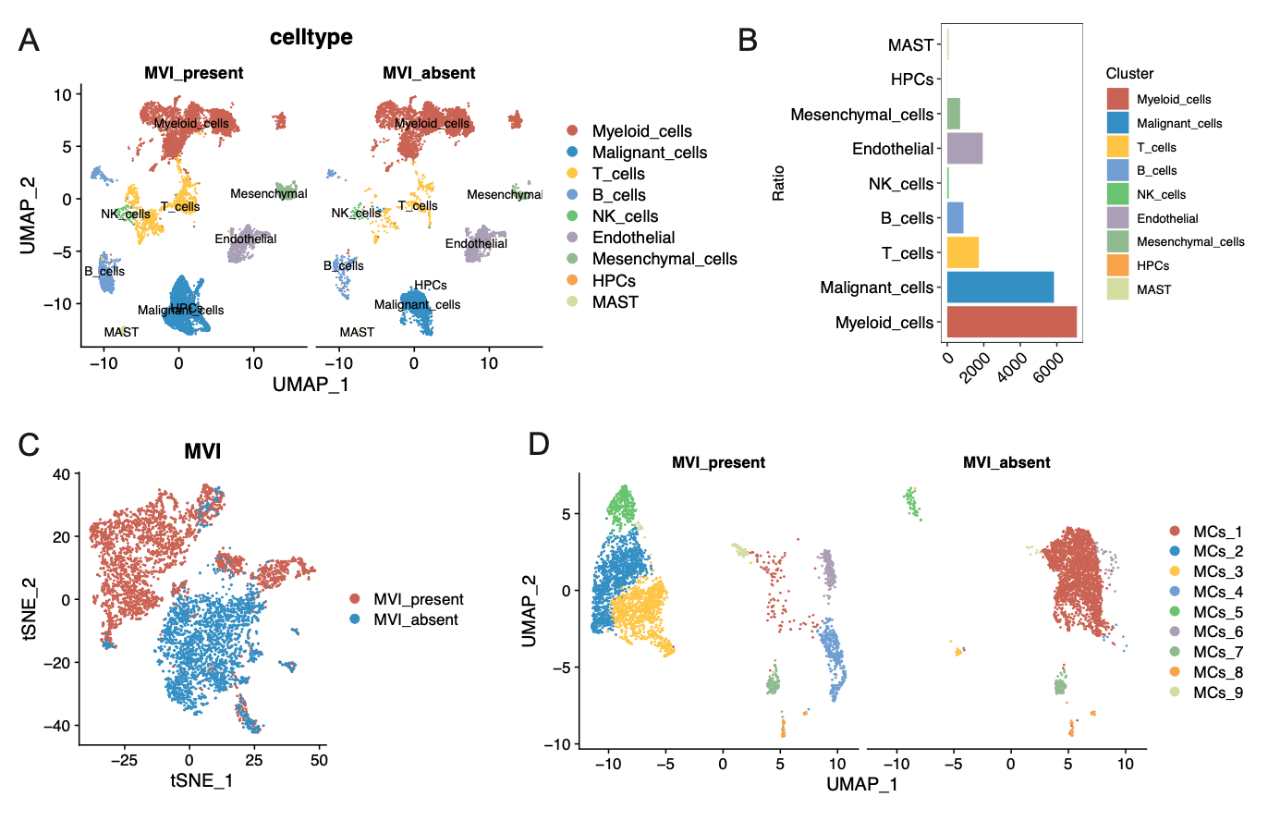


Figure S3: (A) UMAP plot demonstrating the distribution of individual cell subtypes, showing cell distribution according to trans-MVI presence and absence. (B) Stacked bar graph displaying the absolute number of each cell type. (C) Stacked bar graph illustrating the cellular origin of tumor samples. (D) UMAP plot showcasing the segmentation results of malignant cell subgroups, showing cell distribution according to MVI presence and absence.


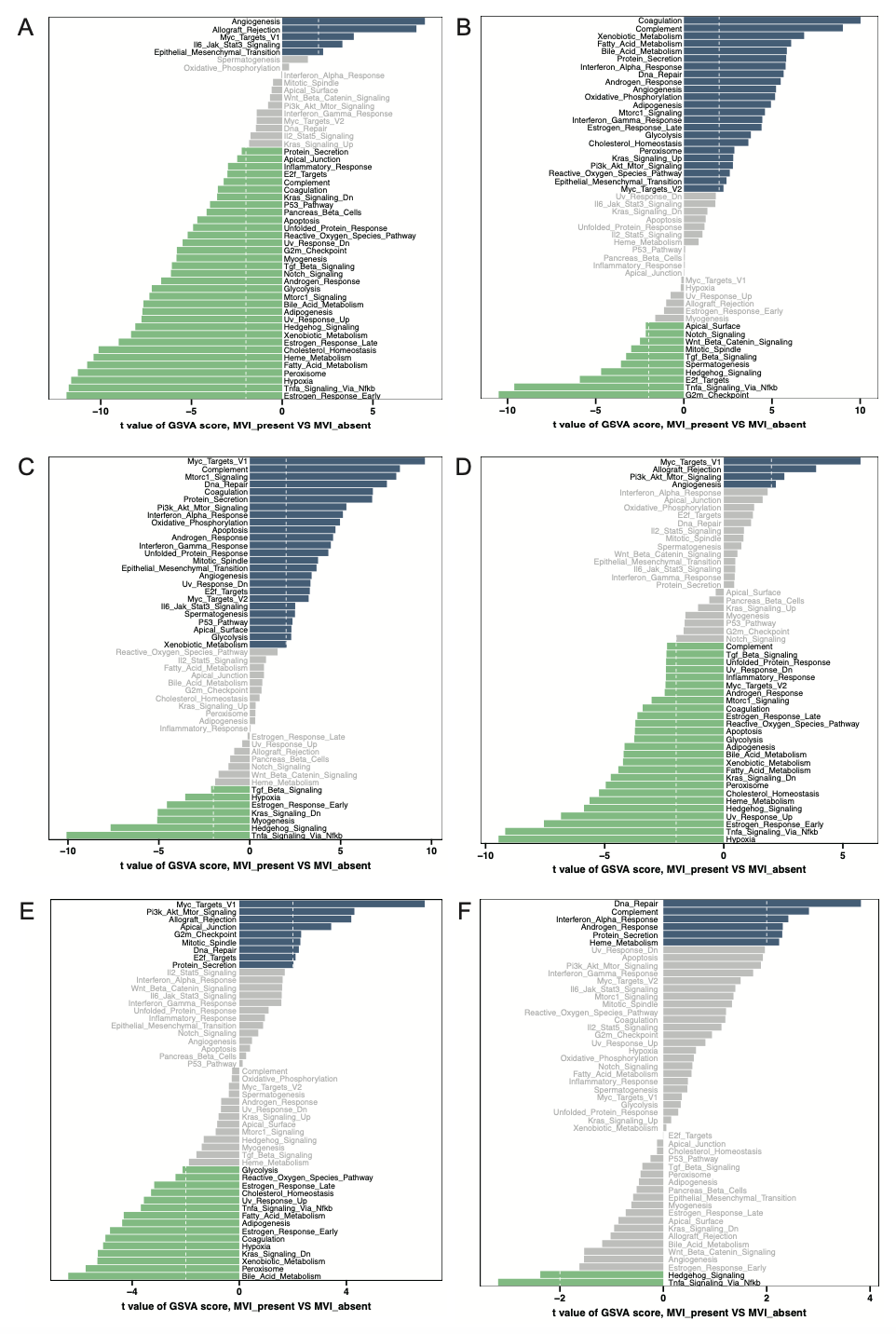


**Supplementary Figure S4:** (A-F) Pathway difference statistics for MVI presence and absence in MCs_1, MCs_3, MCs_5, MCs_7, MCs_8, and MCs_9.


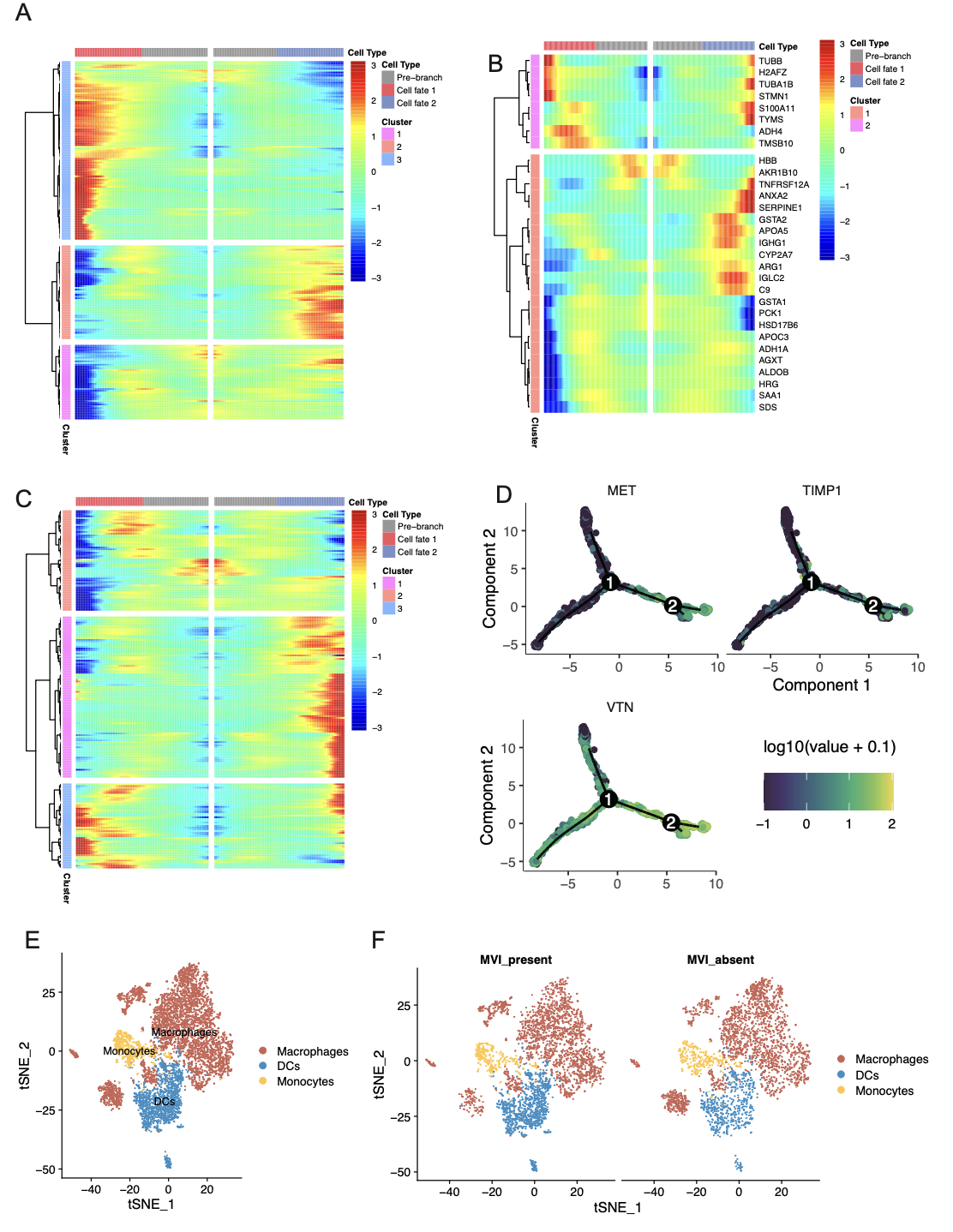


**Supplementary Figure S5:** (A) Pseudo-temporal heatmap displaying changes in the top 200 genes analyzed by BEAM before and after node 1. (B-C) Pseudo-temporal heatmaps showing changes in: (B) the top 30 and (C) the top 200 genes analyzed by BEAM before and after node 2. (D) Expression of MET, TIMP1, and VTN, colored within the trajectory graph. (E) Subdivision of myeloid cell subpopulations. (F) Subdivisions of macrophages, dendritic cells (DCs), and monocytes displayed according to MVI status.


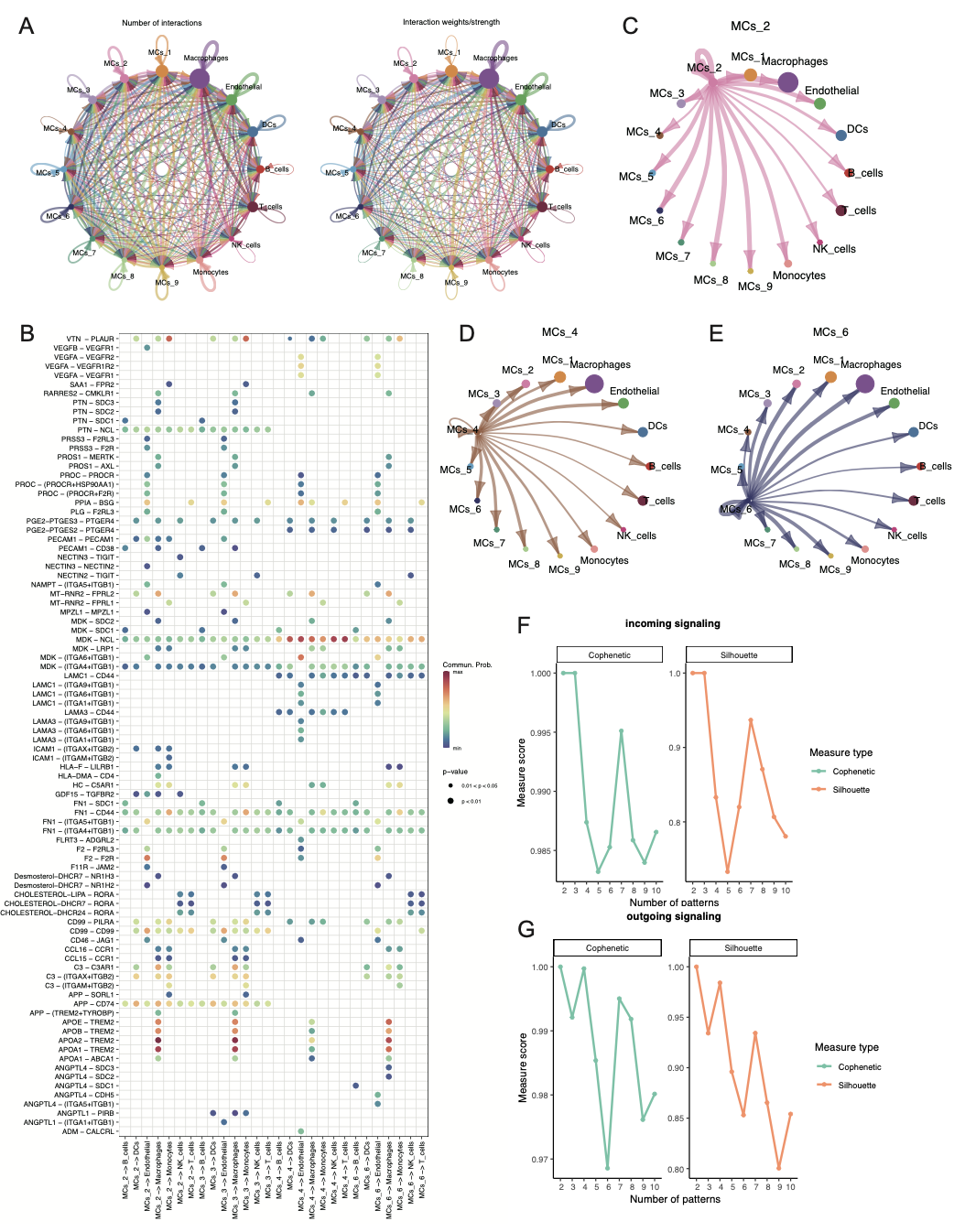


**Supplementary Figure S6:** (A) Circle plot indicating the number (left) and strength (right) of interactions between individual malignant cell subgroups and immune cells. (B) Bubble plots depicting overexpressed ligand-receptor interactions, with bubble size representing the P-value from the alignment test and color indicating the likelihood of interaction. MCs_2, MCs_3, MCs_4, and MCs_6 are shown as signal emitters. (C-E) Visualization of the strength of interactions between MCs_2, MCs_4, and MCs_6 with other cell types. (F-G) Counts of: (F) efferent and (G) afferent signals based on Cophenetic and Silhouette indexing.


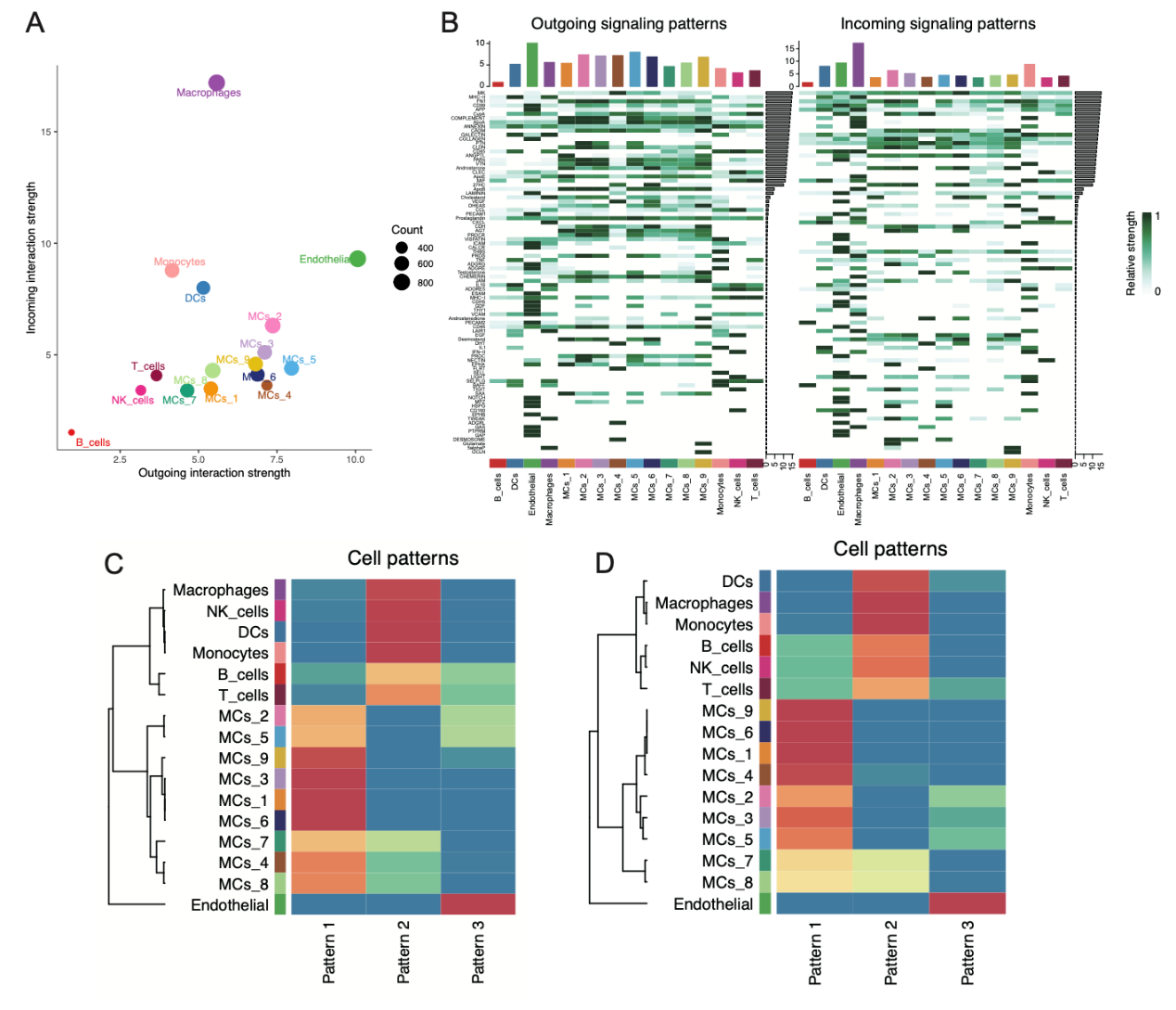


**Supplementary Figure S7:** (A) Dot plot displaying major senders and receivers, with axes representing the total outgoing or incoming communication probabilities associated with each group. Dot size correlates with the number of inferred connections (outgoing and incoming). (B) Heatmap illustrating the efferent or afferent contributions of all signals to different immune cell groups. (C-D) Heatmaps showing the pattern of major: (C) efferent and (D) afferent signals for each cell type.


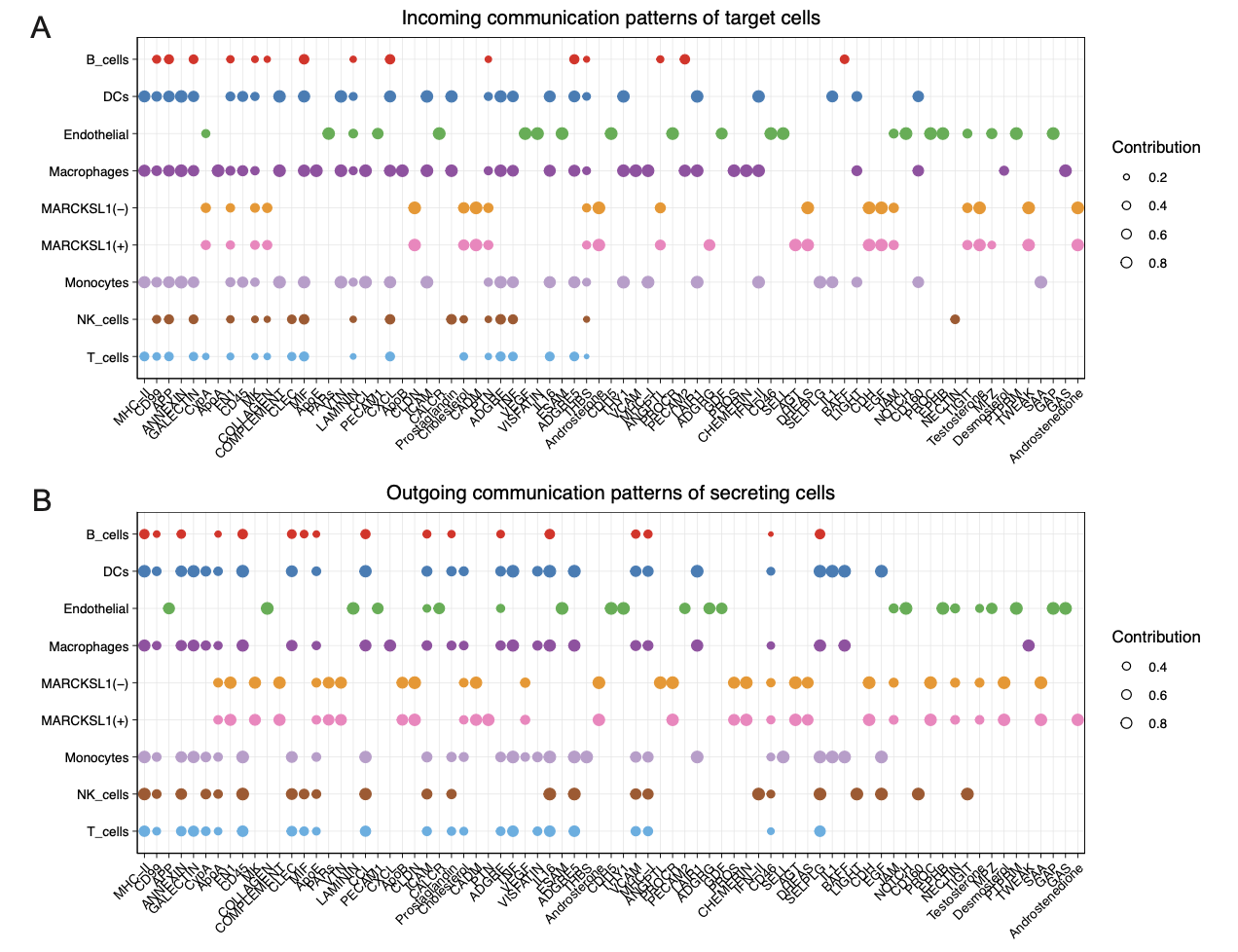


**Supplementary Figure S8:** (A-B) Bubble plots depicting overexpressed ligand-receptor interactions, with bubble sizes representing P-values from alignment tests and colors indicating the likelihood of interaction.
